# Change in general and domain-specific physical activity during the transition from primary to secondary education: a systematic review

**DOI:** 10.1186/s12889-024-18539-1

**Published:** 2024-04-11

**Authors:** Gwennyth E. Spruijtenburg, Femke van Abswoude, Imke L. J. Adams, Sebastiaan W. J. Platvoet, Mark de Niet, Bert Steenbergen

**Affiliations:** 1 Behavioural Science Institute (BSI), Radboud University, Nijmegen, The Netherlands; 2 Institute for Studies in Sports and Exercise, HAN University of Applied Sciences, Nijmegen, The Netherlands

**Keywords:** Child, Adolescent, School transition, Physical activity, Health behaviour, Correlates, Socioecological framework, Systematic review

## Abstract

**Background:**

Knowledge of changes in the domains of physical activity (PA) during the transition period from primary to secondary education and the factors associated with these changes, are prerequisites for the design of effective PA promotion strategies. Therefore, the first aim of this study was to systematically review changes in general, leisure-time, school, transport, work, and home PA across the transition from primary to secondary education. The second aim was to systematically review the individual, social, and physical environmental factors that were associated with these changes.

**Methods:**

Records published up until September 2023 were retrieved from five electronic databases. We included longitudinal and cross-sectional studies that investigated general or domain-specific PA from 2 years before to 2 years after the transition from primary to secondary education. Information on source, study characteristics, sample characteristics, PA, and factors were extracted from the papers included. We reported the direction of change in general and domain-specific PA and the direction of association of the factors with change in general and domain-specific PA.

**Results:**

Forty-eight papers were included in the study. The evidence on changes in PA and associated factors was greatest for general PA. A limited number of the studies investigated the separate domains of leisure-time, school, and transport. Most studies on general and school PA reported a decline in PA, but there were no consistent results for the domains of leisure-time and transport. With respect to the associated factors, evidence was predominantly found for individual factors and to a lesser degree for physical environmental and social environmental factors. None of the factors were consistently associated with changes in general or domain-specific PA during the school transition.

**Conclusions:**

For the design of targeted PA promotion strategies, further studies are warranted to explore changes in the specific domains of PA across the transition from primary to secondary education, especially in the domains of leisure-time, transport, home, and work PA. In addition, the interactions between factors at different socioecological levels to influence changes in PA need to be addressed more in the future.

**Trial registration:**

PROSPERO CRD42020190204.

**Supplementary Information:**

The online version contains supplementary material available at 10.1186/s12889-024-18539-1.

## Background

Physical activity (PA) in children and adolescents can positively influence physical-, mental- and psychosocial health and is also thought to contribute to the development of a long-term active lifestyle [1]. Despite these benefits, most evidence indicates that PA declines with age throughout childhood and adolescence [2, 3]. PA can occur in different domains, such as leisure-time, school, transport, work, or home [4], each of which is likely to be influenced by a distinct set of individual, social- and environmental factors [5]. Major life transitions during childhood and adolescence can have a critical influence on changes in the different domains of PA [6], as they usually coincide with changes in the factors associated with PA.

The transition period from primary to secondary education is a crucial developmental period for maintaining a lifelong active lifestyle [7, 8]. This transition period involves significant changes related to the individual (e.g. the start of puberty), as well as significant changes in the social- (e.g. introduction to new peer groups) and physical environment (e.g. change in distance to travel to school) [8]. The impact of the transition period from primary to secondary education on changes in PA behaviour was shown in two recent reviews [6, 9]. Both of these reviews highlighted that PA behaviours change following the transition from primary school and recognised that the way in which PA changed was dependent upon, for example, the domains and time-periods in which it occurred. Neither of the reviews alluded to domain-specific PA separately. We maintain that a detailed analysis of the different domains is a prerequisite for a thorough insight of the impact of the school transition period. This analysis could provide a more nuanced understanding of PA behaviour compared to the analysis of more general outcomes, such as total daily PA, and thereby determine specific behavioural targets for PA promotion. In this analysis, the individual, social and environmental factors that contribute to domain-specific changes in PA should also be taken into account. In this paper, we reviewed the literature with respect to changes in PA in different domains across the transition from primary to secondary education, together with the factors that are associated with changes in each of the PA domains to build on the insights of previous reviews.

In the review by Chong et al. [9], it was found that total daily PA and PA during specific time-periods (i.e. in-school, after-school, and leisure time) decreased over the school transition period. Gropper et al. [6] also showed that PA generally declined during the transition period from primary to secondary education. However, they also identified studies in which PA increased or did not change. This discrepancy was likely due to differences within studies that were related to domains (e.g. leisure-time and school), intensities (e.g. light, moderate and vigorous), and time-periods (e.g. weekdays or weekends). For example, increases may have been observed in studies that included PA during leisure-time, whereas decreases may have been observed in studies that included PA during school time. However, differences between domains were not explicitly examined in both previous reviews. To our knowledge, only one review examined longitudinal changes in domains of PA in a similar age group (i.e. transition to adolescence at around the age of 13-years-old) [10]. Here it was shown that changes in PA were different for the domains of active transport, organised PA, non-organised PA, and active chores/work. These results emphasize the need to study changes in domain-specific PA, especially during the school transition period, during which an individual’s social and physical environment significantly changes with corresponding changes in PA expected.

For a more detailed understanding of the changes in different domains of PA, additional analyses of the factors that contribute to the changes in PA within/across these domains are required. It is now well established that a number of individual- (e.g. enjoyment of PA), social (e.g. support from parents or friends), and physical environmental factors (e.g. the presence of safe biking paths to and from school) contribute to PA (e.g. [11–14]), and that PA in each domain is likely to be influenced by its own specific factors [5]. Since many of the factors are subject to change over the transition period from primary to secondary education, it is important to also include the possible influence of these factors on domain-specific PA during the school transition period.

In summary, the first aim of this study was to systematically review changes in general and domain-specific PA (i.e. leisure-time, school, transport, work, and home) in the transition from primary to secondary education. The second aim of this study was to systematically review the individual, social and environmental factors that were associated with changes in general and domain-specific PA.

## Methods

This systematic review was conducted in accordance with the Preferred Reporting Items for Systematic Reviews and Meta-Analysis (PRISMA) guidelines [15]. Details of the study protocol were registered in the International Prospective Register of Systematic Reviews (PROSPERO) under the code CRD42020190204.

### Literature search

We performed a systematic literature search using PubMed, Embase, Web of Science, PsycINFO and SPORTDiscus. The initial search was conducted on 3rd April 2020, and updated by the same researchers on 12th September 2023. The search strategy included medical subject headings and free-text terms for the following themes: physical activity, transition, primary or secondary school, and participants (i.e. children and/or adolescents). No publication filters were applied. See Additional File 1 for details of the search strategy.

### Inclusion and exclusion criteria

Inclusion and exclusion criteria are presented in Table 1. We included longitudinal and cross-sectional studies that investigated general or domain-specific PA, before, as well as after the transition from primary to secondary education. Both longitudinal and cross-sectional studies were included because we wanted to provide a very broad picture of the available evidence on the impact of the school transition period. Although cross-sectional studies obviously do not provide evidence on longitudinal changes in PA, we chose to include them to ensure that potentially relevant information was not lost. Only studies with children and adolescents ranging in age between six- and 18-years-old, and without a specific disease, were included as participants. We chose a relatively broad age range to ensure inclusion of all potentially relevant papers. In addition, the combination of the inclusion criteria regarding exposure (i.e. transition from primary to secondary education) and study type (i.e. PA data within 2 years before and after the school transition) automatically selected the relevant age period. No restrictions were imposed on publication date or country of the study in the initial search. However, in the updated search, we applied a ‘date of publication’ filter, so that only papers published after the initial search were included. Studies that were not published in a scientific journal or not published in English were excluded.

**Table 1:** Inclusion and exclusion criteria

	Inclusion criteria	Exclusion criteria
Setting	All countries	
Participants	Children and adolescents aged between six- and 18-years-old.	Children and adolescents with a specific disease.
Exposure	The transition from primary to secondary education.	
Outcomes	Studies that reported at least one measure of general or domain-specific PA, both objective and self-report, for example: MVPA, total PA, sports participation, active transport, PA level at school, etc..	Studies that reported only on sedentary behaviour but not on PA.
Study type	Longitudinal and cross-sectional studies, both quantitative and qualitative, which included PA data within 2 years before and 2 years after the transition.	
Publication type	Journal article.	Other publication types: Conference abstract, study protocol, report, dissertation, book, professional journal.
Publication year	All publication years	None.
Language	English.	All other languages.

### Study selection

**Fig. 1:**
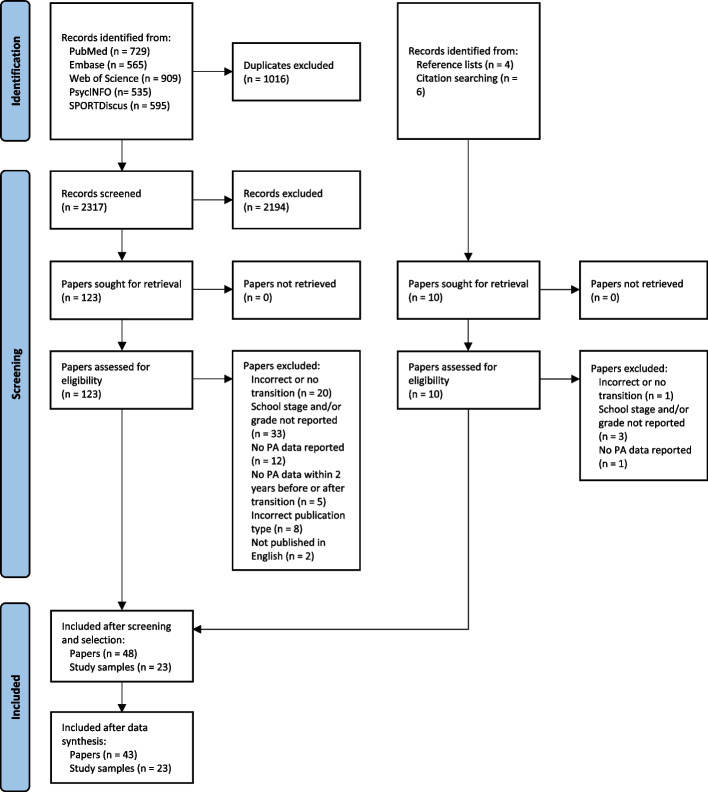
PRISMA flow diagram of the systematic literature search

Records generated through the initial search were exported to EndNote X9 and duplicates were removed. Then, the title and abstract screening approach was piloted: One reviewer (GS) screened the first 500 results and discussed the approach with two other reviewers (FvA and BS). After consensus was reached, the remainder of titles and abstracts were screened for eligibility by two reviewers (GS and FvA). Of the potentially eligible articles, the full text was retrieved and assessed by the two reviewers for final inclusion. Any doubts or disagreements were resolved through a process of critical debate between the two reviewers. If consensus could not be reached, a third reviewer (BS) was consulted. Next, one reviewer (GS) hand-searched reference lists of included papers and carried out citation searches to identify additional full texts to be considered for inclusion. The additional full texts were assessed by two reviewers (GS and FvA) and discussed with the third reviewer (BS) in cases of doubt or disagreement. The screening and selection process of the updated search was performed by one reviewer (GS). The PRISMA flow diagram is shown in Fig. 1.

### Quality assessment

Quantitative papers were assessed for methodological quality using a modified version of the Quality Assessment Tool for Observational Cohort and Cross-Sectional Studies published by the National Heart, Lung and Blood Institute (NHLBI) [16]. After a consensus between the reviewers (GS and FvA), multiple modifications to the original version were made as follows:

1. In all questions regarding exposure and/or outcome measures, “exposure (measure)” was changed to “independent variable”, and “outcome (measure)“was changed to “dependent variable”.
2. A question to quantify the duration of the timeframe between the assessment of independent and dependent variables was added.
3. Questions regarding definition, validity and reliability of independent and dependent variables were reorganised into one regarding definition and one regarding validity and reliability.
4. Questions regarding repeated exposure assessment and blinding of outcome assessors were deleted.
5. The question regarding confounding variables was changed into an open question.

The modified tool consisted of 15 questions, 13 of which could be scored with ‘Yes’, ‘No’, ‘Not Applicable’, or ‘Not Reported’. Questions 7 and 15 remained as open questions. Qualitative papers were assessed for methodological quality using the Joanna Briggs Institute (JBI) Critical Appraisal Checklist for Qualitative Research [17]. This tool consists of 10 items, each of which could be scored with ‘Yes’, ‘No’, ‘Unclear’ of ‘Not applicable’.

Two reviewers (GS and FvA) independently scored each quantitative and qualitative paper. Scores were then compared and discussed until consensus was reached. After that, each paper was given a percentage score based on the number of applicable criteria (i.e. items that were scored ‘Yes’, ‘No’, ‘Not reported’, or ‘Unclear’) that were considered fulfilled (i.e. items that were scored ‘Yes’). Multiple papers using the same study sample were assessed separately, as they could contribute to the analysis of different outcomes.

### Data extraction

Data extraction was done by using a Microsoft Excel spreadsheet developed by three reviewers (GS, FvA and BS). The data extraction form was piloted on 10 randomly selected included studies by one reviewer (GS), after which any uncertainties were discussed with the two other reviewers (FvA and BS), and improvements were made upon agreement between the reviewers. One reviewer extracted data from all included articles (GS).

For the articles included details on 1) source, 2) study characteristics, 3) sample characteristics, 4) PA, and 5) individual, social and physical factors were extracted. Details on source included authors, year, country, and study name. Details on study characteristics included study design, frequency and duration of follow-up(s), and school transition grade/year. Details on participants characteristics included sample sizes at baseline and follow-ups, age, and gender. Details on PA included assessment tools, outcomes, domain, intensity, duration, frequency, results at baseline and follow-up (i.e. mean and SD), and reported change (and significance) in PA. Details on factors included assessment tools, outcomes, and results on associations with change in PA.

### Data synthesis

A descriptive synthesis was developed separately for both research questions by one reviewer (GS) and checked by a second reviewer (FvA). Disagreements were resolved through consensus. No meta-analysis was conducted.

#### Changes in PA

Only quantitative papers were found eligible for the first research question. These papers reported PA in various ways. To enable comparison of results across studies, PA outcomes were first grouped into six domains (i.e. general, leisure-time, school, transport, work, and home), and then into smaller groups with respect to PA outcomes. These smaller groups were developed after data extraction and summarised the results of multiple related outcomes (hereafter, we refer to such a group as a ‘PA synthesis unit’). PA outcomes of general PA were grouped based on their intensity level (e.g. MVPA, LPA). Number of steps was included as an extra synthesis unit in this domain. Synthesis units in the school domain were based on the types of PA that can take place during the school day (i.e. general, recess/lunch, physical education (PE), extracurricular). Leisure-time synthesis units were based on whether the PA outcome could be classified as organised and/or non-organised. Synthesis units in the domain of transport were based on the destination of travel (e.g. general, school, leisure-time destinations). The only synthesis unit in the home domain consisted of common activities of daily life (CADL). PA outcomes in all domains were also grouped according to whether they were measured objectively (e.g. accelerometer, pedometer) or subjectively (e.g. questionnaire). Table 2 lists the PA synthesis units per PA domain and examples of PA outcomes included in each PA synthesis unit.

**Table 2:** PA synthesis units and examples of PA outcomes investigated

PA synthesis units	Examples of PA outcome
***General***	
All intensities (objective)	PA in minutes per hour and metabolic equivalent (different time segments)
All intensities (subjective)	PA in minutes per day and PA level on a 5-point scale (different time segments)
MVPA (objective)	MVPA in minutes per hour and minutes per day (different time segments)
MVPA (subjective)	MVPA in number of 30-minute blocks per day
LPA (objective)	LPA in minutes per day (different time segments)
MPA (objective)	MPA in minutes per day (different time segments)
VPA (objective)	VPA in minutes per day (different time segments)
VPA (subjective)	VPA in number of 30-minute blocks per day
Number of steps (objective)	Number of steps per day (different time segments)
***School***	
General school (objective)	School PA in minutes per hour and minutes per day (different intensities)
General school (subjective)	School PA level on a 5-point scale
Recess/lunch (objective)	Recess/lunch PA in percentage of time (different intensities)
Recess/lunch (subjective)	Recess/lunch PA level on a 5-point scale
Physical education (subjective)	Physical education PA level on a 5-point scale and participation (yes/no) in physical education
Extracurricular PA (subjective)	Extracurricular PA in minutes per day and participation (yes/no) in competitive activities in school
***Leisure-time***	
Organised and non-organised sport (objective)	Sport in minutes per day (different intensities)
Organised and non-organised sport (subjective)	Sports during leisure in minutes per day
Organised sport (subjective)	Participation (yes/no) in competitive activities out of school and organised non-competitive activities
Organised and non-organised PA (subjective)	PA level during leisure time in minutes per day and on a 5-point scale
Non-organised PA (objective)	Active leisure in minutes per day
Non-organised PA (subjective)	Participation (yes/no) in non-organised activities
***Transport***	
General active transport (objective)	Active transport in minutes per day (different intensities and time segments)
Active transport to school (subjective)	Active transport to school in minutes per day and travel mode to school
Active transport during leisure (subjective)	Active transport during leisure in minutes per day and travel mode to leisure time destinations
***Home***	
Common activities of daily life (objective)	CADL in minutes per day

For each study sample, we reported change in PA (i.e. negative, no change, positive or unclear) maximum once per PA synthesis unit (i.e. group of PA outcomes). However, during the processes of data extraction and grouping of PA outcomes, we noticed that:

1. Multiple papers using the same study sample would report data on the same PA synthesis unit.
2. One paper would report data on multiple PA outcomes of the same PA synthesis unit.
3. One paper would report multiple results for 1 PA outcome (i.e. subgroup analysis, multiple timeframes, adjusted analysis).

**Fig. 2:**
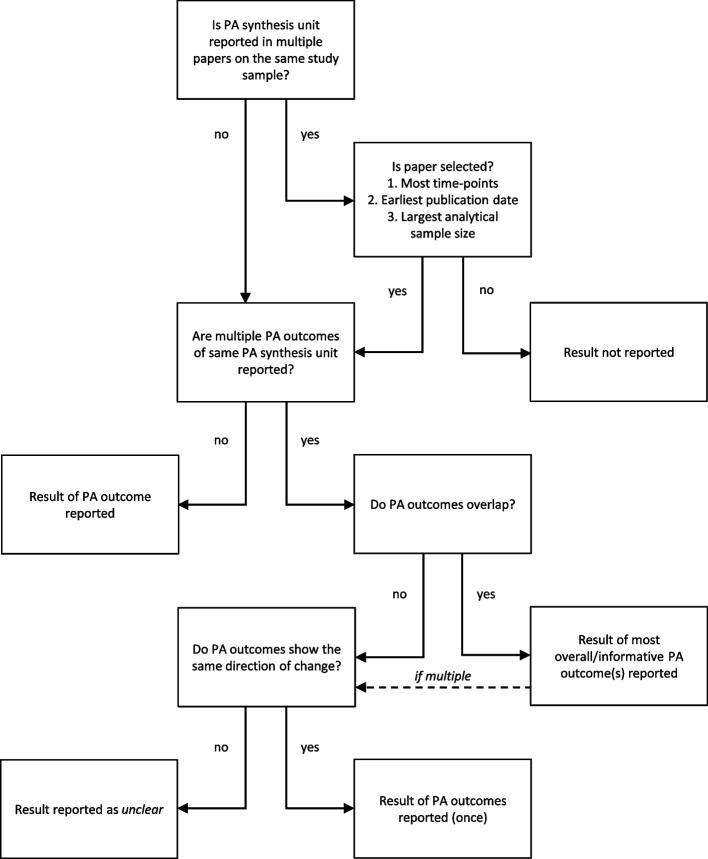
Decision strategies for the selection of papers and PA outcomes

**Fig. 3:**
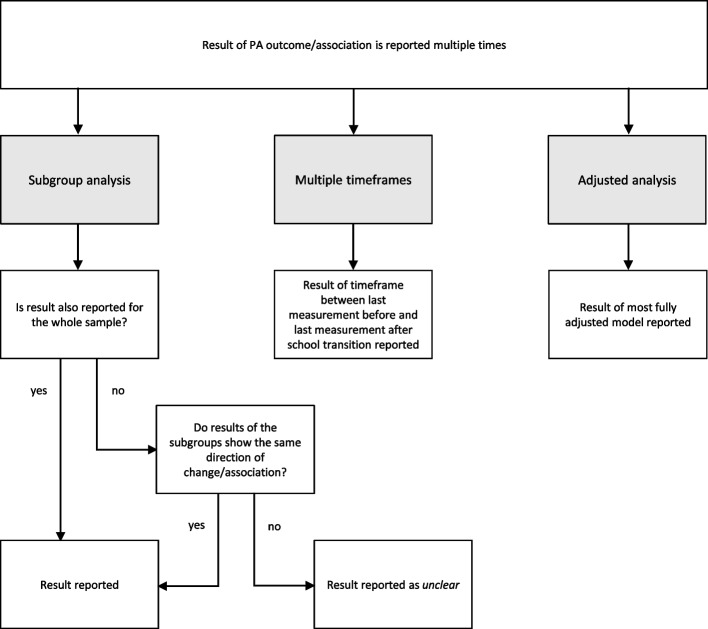
Decision strategies for the selection of results

We developed several decision strategies to 1) select one paper per PA synthesis unit for each study sample, 2) select the least possible PA outcomes per PA synthesis unit for each paper, and 3) select one result for each PA outcome (see Figs. 2 and 3 for all decision strategies).

Examples of overlapping outcomes are MVPA and after-school MVPA, and active transport in duration (minutes/day) and active transport in binary response (yes/no). In these examples, MVPA and active transport in duration are the most overall and informative outcomes, respectively. Examples of non-overlapping outcomes are weekday MVPA and weekend MVPA, and walking to school and bicycling to school.

If the statistical significance of change in PA of a selected paper was not stated but descriptive statistics were reported, online calculators (with default settings) were used to calculate the significance of change [18]. A dependent t-test was performed for one paper [19]. For other papers, independent sample t-tests (pooled variance) [20–26], and a two sample proportion test [20] were performed as paired data were not available.

Some PA outcomes did not conform to the categorisation of PA outcomes in domains: PA outcomes concerning specific locations and contexts, and PA outcomes that were rest categories in the original paper (e.g. *other* PA and PA in *other* locations). PA outcomes concerning specific locations and contexts could not be grouped in domains because activities at a specific location or in a specific context do not necessarily always occur in the same domain. For example, activities at school grounds could fall within the school domain, as well as within the leisure-time domain. As our aim was broad (to systematically review as much evidence as possible on changes in PA across the transition from primary to secondary education), we decided to describe results on location- and context-specific PA separately from the results on general and domain-specific PA. However, the PA outcomes that were rest categories in the original paper were excluded from our synthesis.

#### Factors associated with changes in PA

Factors were first grouped according to level of influence (i.e. individual, social, and physical environmental) and then in smaller groups of related factors. Again, these smaller groups were developed after data extraction. Individual factors were grouped in demographic information, physical characteristics, motivation and goals, self-concept, beliefs about capabilities, and beliefs about consequences. Social environmental factors were grouped according to the source of support (e.g. parents, friends, teacher). Physical environmental factors were grouped with respect to home, neighbourhood and school environments. Table 3 lists the groups of factors and examples of factors included in each group.

**Table 3:** Groups of factors and examples of factors investigated

Groups of factors	Examples of factors
***Individual factors***	
Demographic	Gender, race, socioeconomic status, family size
Physical characteristics	Weight status, maturity
Motivation and goals	Enjoyment, motivation, goals, amotivation
Self-concept	Self-schema, perceived aggressive disposition, physical self-perceptions
Beliefs about capabilities	Self-efficacy, perceived competence
Beliefs about consequences	Perceived benefits, perceived barriers
***Social environmental factors***	
Parental influence	Parental support, parental rules and norms, parents’ PA habits
Peer influence	Peer support or pressure, friend’s PA habits, peer-created motivational climate
Teacher influence	Teacher support
***Physical environmental factors***	
Home equipment	PA and sedentary equipment at home
Neighbourhood environment	Perceived and observed neighbourhood environment
School environment	Physical education time, extracurricular PA promotion, school commute environment

**Fig. 4:**
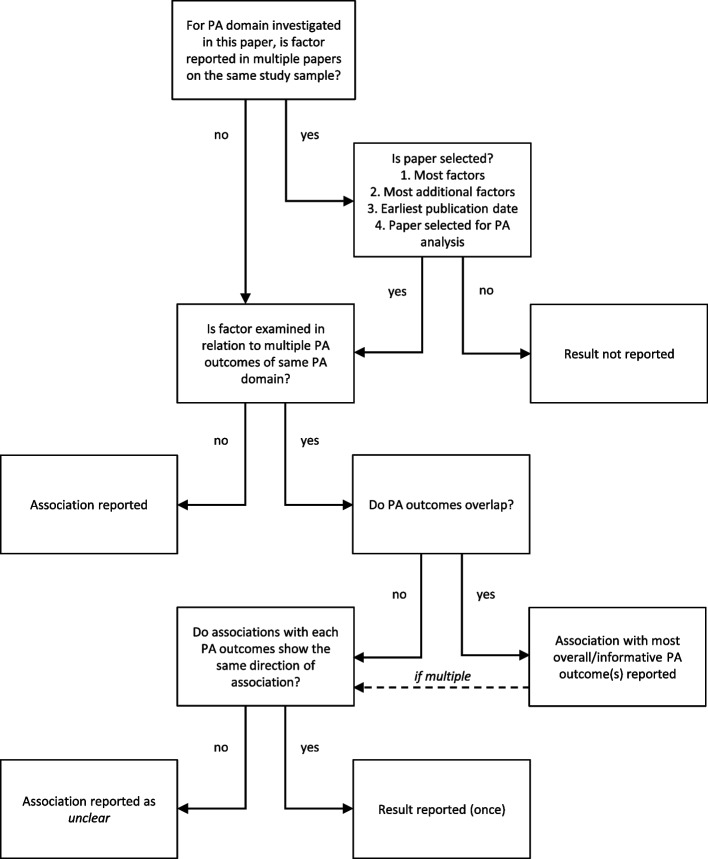
Decision strategies for the selection of papers and associations

For each study sample, the direction of association of one factor with change in PA (i.e. negative, null or positive association, or unclear) was reported a maximum of once per PA domain. We noticed in earlier processes of the review that 1) multiple papers using the same study sample would report data on the same factor and PA domain, 2) one paper would report data on the same factor and multiple PA outcomes of the same PA domain, and 3) one paper would report multiple results for one association (i.e. subgroup analysis, multiple timeframes, adjusted analysis). We developed several decision strategies to 1) select one paper per factor and PA domain for each study sample, 2) select the least possible associations per PA domain for each paper, and 3) select one result for each association (see Figs. 3 and 4 for all decision strategies).

Factors that were investigated in association with PA outcomes which did not conform the categorisation of PA outcomes in domains (i.e. PA outcomes concerning specific locations and contexts), and factors retrieved from qualitative papers were described in an additional analysis.

## Results

### Study characteristics

In total, 43 papers were included [19–60] and five papers that reported data that was already reported in another paper of the same study sample [61–65] were excluded (see Fig. 1). Additional File 2 (Table S1) presents decisions for the selection of the PA outcomes and factors that were reported in our review. Additional File 3 (Table S1) presents details of the studies of selected PA outcomes and factors. Papers that included prospective designs predominated in the review (*n* = 37). Cross-sectional designs were used in three papers and retrospective qualitative designs were used by three papers. Papers with prospective designs investigated change in general or domain-specific PA (*n* = 26), factors associated with change in PA (*n* = 19) and change in PA locations and contexts (*n* = 4). Papers with cross-sectional designs investigated differences in PA (*n* = 3) and factors associated with change in PA (*n* = 2). Studies with retrospective qualitative designs investigated factors associated with change in PA (*n* = 3).

### Quality assessment

Of the applicable criteria in all papers (i.e. quantitative and qualitative), 72% were considered fulfilled. Percentage scores ranged from 40 to 92%. Criteria that were most often scored with ‘No’ in the 40 quantitative papers concerned the sample size justification (Item 5) and follow-up rate (Item 14). Criteria that were often scored with ‘Not reported’ concerned the study population (Item 3) and timeframe (Items 7 and 8). Confounding variables that were measured regularly were socioeconomic status and gender. Criteria that were scored with ‘No’ in two of the three qualitative papers concerned the congruity between research methodology and interpretation of results (Item 5), statement locating the researcher culturally or theoretically (Item 6) and influence of the researcher on the research, and vice-versa (Item 7). Scores for each of the items can be found in Additional File 4 (Tables S1 and S2).

### Change in PA

Results of the analysis on changes in general and domain-specific PA across the transition from primary to secondary education can be found in Table 4 and Additional File 5 (Tables S1-S3). Twenty-nine papers on 22 different study samples were included in this part of our review. A total of 66 synthesis units (i.e. changes in a group of PA outcomes) were reported. The mean number of synthesis units per study sample was 2.9 and varied from 1 to 11. Study samples that contributed to more than one tenth of the included synthesis units were two samples of Belgian schoolchildren (*n* = 11 and *n* = 7), the Transitions and Activity Changes in Kids (TRACK) sample (*n* = 6), and a sample of Australian schoolchildren (*n* = 6). General PA was the most investigated PA domain (*n* = 35), followed by school (*n* = 14), leisure-time (*n* = 9), transport (*n* = 7) and home (*n* = 1). Changes in groups of objectively measured PA outcomes (*n* = 33) were reported as many times as changes in groups of subjectively measured PA outcomes (*n* = 33). Evidence on change in PA was mixed between and within PA domains, with no consistent change of direction observed. However, a significant decrease was reported more often (30/66 synthesis units) than no change (16/66), or an increase (11/66). In nine out of 66 synthesis units, the direction of change in PA was unclear (see Additional File 5 for detailed information on all results that were classified as unclear).

**Table 4:** Summary of changes in PA synthesis units

PA domain	PA synthesis units	–	=	+	unclear	total
General	All intensities / Total PA (objective)	2	2	0	0	4
	All intensities / Total PA (subjective)	4	3	1	1	9
	MVPA (objective)	6	1	2	1	10
	MVPA (subjective)	1	0	0	0	1
	LPA (objective)	4	1	0	0	5
	MPA (objective)	0	0	1	0	1
	VPA (objective)	0	0	1	0	1
	VPA (subjective)	0	1	0	0	1
	Number of steps	1	2	0	0	3
	Total	18	10	5	2	35
School	General school (objective)	3	0	0	0	3
	General school (subjective)	1	0	0	0	1
	Recess/lunch (objective)	0	0	0	1	1
	Recess/lunch (subjective)	2	0	0	0	2
	Physical Education (subjective)	0	1	2	1	4
	Extracurricular PA (subjective)	2	1	0	0	3
	Total	8	2	2	2	14
Leisure-time	Organised and non-organised (objective)	0	0	1	0	1
	Organised and non-organised (subjective)	2	0	0	2	4
	Organised (subjective)	0	2	0	0	2
	Non-organised (objective)	0	1	0	0	1
	Non-organised (subjective)	0	0	1	0	1
	Total	2	3	2	2	9
Transport	General active transport (objective)	1	0	0	1	2
	Active transport to school (subjective)	1	0	2	1	5
	Active transport during leisure (subjective)	0	0	0	1	1
	Total	2	0	2	3	7
Home	Common activities of daily life (objective)	0	1	0	0	1
	Total	0	1	0	0	1

Change is reported as negative (−), no change (=), positive (+) or unclear

General PA was investigated in 22 papers for 19 different study samples (see Table 4 and Additional File 5). Among general PA, PA outcomes representing all intensities (13/35 synthesis units) and MVPA (11/35) were the most investigated intensity levels. Evidence on PA outcomes representing LPA (5/35), MPA (1/35), VPA (2/35) and number of steps (3/35) was very scarce. Evidence of limited consistency showed that, overall, general PA declined across the transition from primary to secondary education (18/35). Less evidence was observed for no change (10/35) and an increase (5/35) in general PA. The direction of change was unclear in two out of 35 synthesis units.

School PA was investigated in 11 papers for 10 study samples. Evidence for each group of PA outcomes within the school domain was limited, with four, three, four and three synthesis units reported for general school, recess/lunch, physical education (PE) and extracurricular PA, respectively. General school (4/4), recess/lunch (2/3) and extracurricular PA (2/3) showed mainly declines over the school transition period. Evidence on physical education PA never showed any declines. Instead, results indicated increases (2/4) and no change (1/4) for physical education PA. In two out of 14 synthesis units, evidence on change in PA was unclear.

Leisure-time PA was investigated in seven papers for seven study. Evidence on leisure-time PA was very scarce and inconsistent. Studies reported declines (2/5) and an increase (1/5) for the combination of organised and non-organised PA, no change (2/2) for organised PA and no change (1/2), and an increase (1/2) for non-organised PA. The direction of change in PA was unclear in two out of nine synthesis units.

Transport PA was investigated in eight papers for seven study samples. Again, evidence was scarce (seven synthesis units) and inconsistent. Results indicated a decline (1/2) for general active transport and a decline (1/4) and increases (2/4) for active transport to school. In three out of eight synthesis units, the direction of change was unclear.

Only one study investigated home PA. Home PA in this study did not change over the school transition.

#### Additional results

PA locations were investigated in four quantitative papers for two different study samples [33, 41, 55, 58]. Although it was shown that the majority of children of the TRACK sample stayed physically active in the same locations between primary and secondary school, some did change the variety of the locations in which they were physically active [33]. These particular children were most likely to decrease the number of their PA locations [33, 58]. In addition, declines were observed for the amount of PA at some specific locations (e.g. home, neighbourhood, school grounds, sports grounds [41, 55]. However, the results of previous studies also suggest that changes in the amount of PA at specific locations were dependent on the intensity of PA (e.g. LPA, MVPA) and daily time periods (i.e. weekend, before school, during school, after school).

One paper investigated the social and temporal contexts of common physical activities [58]. The social contexts of PA generally showed a shift from social groups (e.g. with one or several others) toward group contexts which are likely to be more structured (e.g. class and team), whereas the temporal contexts of PA (e.g. during and outside of school) did not change across the transition from primary to secondary education.

### Factors associated with change in PA

Results of the analysis on factors associated with change in PA can be found in Tables 5 and 6 and Additional File 6 (Tables S1-S4). Factors of 20 papers on 12 different study samples were included in our review. In total, 201 synthesis units (i.e. associations between one factor and change in 1 PA domain) were reported. The mean number of synthesis units per study sample was 16.8 and varied from 1 to 102. Study samples that contributed to a large proportion of the included synthesis units, approximately one fifth to half, were the TRACK sample (*n* = 40) and a sample of Belgian schoolchildren (*n* = 102). Factors were most often investigated in association with general PA (*n* = 116), followed by transport (*n* = 40), leisure-time (*n* = 31), school (*n* = 13) and home (*n* = 1). The most investigated level of influence was the individual level (*n* = 92). Physical environmental (*n* = 70) and social environmental (*n* = 39) followed thereafter. No evidence of association was observed in almost all synthesis units (170/202). Only a small number of associations between a factor and change in 1 PA domain were significant (9/201) or significant only for specific PA outcomes within the PA domain and/or for specific subgroups (23/201) (See Additional File 6 for detailed information on all results that were classified as unclear).

**Table 5:** Summary of associations between demographic factors and change in PA

		General		Leisure-time		Transport		School		Home		Total
Level of influence	Group of factors	n.s.	sig.	n.s.	sig.	n.s.	sig.	n.s.	sig.	n.s.	sig.	
Individual	Demographic factors	7	0	2	0	3	0	4	0	1	0	17

Association is reported as not significant (n.s.) or significant (sig.)

**Table 6:** Summary of associations between factors and change in PA

Level of influence	Group of factors	General				Leisure-time				Transport				School				Total
		n.s.	+	–	u	n.s.	+	–	u	n.s.	+	–	u	n.s.	+	–	u	
Individual	Physical characteristics	5	0	0	0	0	0	1	0	0	0	0	0	1	0	0	0	7
	Motivation and goals	10	2	0	2	1	0	0	0	1	0	0	0	0	0	0	0	16
	Self-concept	4	1	0	0	0	0	0	0	0	0	0	0	1	0	0	0	6
	Beliefs about capabilities	6	1	0	0	0	0	0	1	1	0	0	0	0	0	0	0	9
	Beliefs about consequences	12	0	0	2	10	0	0	1	8	0	0	4	0	0	0	0	37
	Total	37	4	0	4	11	0	1	2	10	0	0	4	2	0	0	0	75
Social environmental	Parental influence	14	1	0	0	2	0	0	1	2	0	0	1	0	0	0	0	21
	Peer influence	12	0	0	2	1	0	0	0	1	0	0	0	1	0	0	0	17
	Teacher influence	1	0	0	0	0	0	0	0	0	0	0	0	0	0	0	0	1
	Total	27	1	0	2	3	0	0	1	3	0	0	1	1	0	0	0	39
Physical environmental	Home environment	4	0	0	0	0	0	0	0	0	0	0	0	0	0	0	0	4
	Neighbourhood environment	17	1	0	2	9	0	0	1	9	0	0	2	0	0	0	0	41
	School environment	8	0	1	1	1	0	0	0	6	0	0	2	4	1	0	1	25
	Total	29	1	1	3	10	0	0	1	15	0	0	4	4	1	0	1	70

Association is reported as not significant (n.s.), positive/protective against declines (+), negative/contributing to declines (−) or unclear

#### Individual factors

Individual factors of 14 papers for 10 different study samples were included in our review (See Tables 5 and 6 and Additional File 6). General PA was the domain within which most associations were investigated (52/92 synthesis units), followed by transport (17/92), leisure-time (16/92), school (6/92), and home PA (1/92). No evidence of association between individual factors and change in PA was observed in the majority of synthesis units (77/92).

Demographic factors were investigated in eight papers for six different study samples. Among this group of factors, gender was the most investigated factor (12/17). Socioeconomic status (3/17), family characteristics (1/17), and race (1/17) followed thereafter. A lack of association between demographic factors and change in PA was consistently observed for the included synthesis units (17/17). Gender was not associated with change in general (4/4), leisure-time (2/2), transport (2/2), school (3/3) and home PA (1/1). Socioeconomic status was not associated with change in general (1/1), transport (1/1) and school PA (1/1). Family characteristics were not associated with change in general (1/1). Race was not associated with change in general PA (1/1).

Physical characteristics were investigated in two papers for two study samples. BMI was examined in 3 out of 7 synthesis units in this group, whereas body mass (1/7), skinfolds (1/7), waist circumference (1/7), and maturation (1/7) were each examined once. Again, evidence of no association was observed in the majority of synthesis units (6/7). BMI was not associated with change in general (1/1) and school PA (1/1), but, in one study sample, BMI was associated with change in leisure-time PA (1/1). This study indicated that children with overweight had larger declines in leisure-time PA than children with a typical weight. Body mass, skin folds, waist circumference and maturation were not associated with change in general PA.

Motivation and goals were investigated in six papers for five study samples. Factors investigated in this group were enjoyment of PA (6/16) and physical education (1/16), motives for PA (e.g. enjoyment, competence, social) (5/16) and motivational regulations (e.g. intrinsic, external, amotivation) (4/16). Papers that reported a lack of association between motivation and goals and change in PA predominated (12/16). Enjoyment of PE, motives and motivational regulations were not associated with change in general PA (10/10). Evidence was less consistent for the four included synthesis units between enjoyment of PA and change in general PA: In two study samples, enjoyment was associated with lower declines in PA in the whole group, while in two other study samples, evidence was unclear. No evidence of association between enjoyment of PA and leisure-time (1/1) as well as transport PA (1/1) was observed.

Self-concept was investigated in three papers for three study samples. Evidence on self-concept was limited in six factors (i.e. body attractiveness, muscular strength and development, overall physical self-worth, physical condition, self-schema, and perceived aggressive disposition) that were each investigated once in association with change in PA. One result of significant association was observed for physical condition and general PA. No evidence of significant associations of body attractiveness, muscular strength and development, overall physical self-worth and self-schema with change in general PA was observed. Perceived aggressive disposition was not associated with school PA.

Beliefs about capabilities were investigated in six papers for five study samples. Among this group, self-efficacy (5/9) and perceived competence (4/9) were the factors investigated in association with change in PA. No evidence of association was observed in the majority of synthesis units (7/9). Self-efficacy was not associated with change in general (3/3) and transport PA (1/1). Evidence on the association between self-efficacy and leisure-time PA was unclear (1/1). Perceived competence was generally not associated with change in general PA (3/4). However, one result of a positive association between perceived competence and change in general PA was observed (1/4).

Beliefs about consequences were investigated in four papers for three different study samples. This group mainly consisted of perceived benefits (e.g. fun, health, meeting friends) (18/37), and barriers (e.g. not liking sport, not being good at sports, lack of time) (17/37). Other factors in this group were benefits-barriers ratio (1/37) and perceived outcomes of regular PA (1/37). A lack of evidence of association between beliefs about consequences and change in PA was observed in 30 out of 37 synthesis units. Perceived benefits were not associated with change in general (6/6), leisure-time (6/6) and transport PA (4/6). Perceived barriers were also not associated with change in general (5/7), leisure-time (4/5) and transport PA (3/5). No evidence of association between perceived outcomes of regular PA and general PA (1/1) and between benefits-barriers ratio and transport PA (1/1) were observed. In seven out of 37 synthesis units, evidence of association between beliefs about consequences and change in PA was unclear.

#### Social environmental factors

Social environmental factors of eight papers for seven different study samples were included in our review (see Tables 5 and 6 and Additional File 6). General PA was the domain within which most synthesis units were investigated (30/39), followed by leisure-time (4/39), transport (4/39), and school (1/39). No evidence of association between social environmental factors and change in PA was observed in the majority of synthesis units (34/39).

Parental influence was investigated in four papers for four study samples. Among this group of factors, parental support (9/21), parental social norm (4/21), parental trust in child’s ability to be physically active (3/21), parents’ enjoyment of (1/21) and participation in PA (2/21), and parental rules about sedentary behaviour (2/21) were investigated. No evidence of association between parental influence and change in PA was consistently observed among included synthesis units (18/21). Parental support was not associated with general PA (6/7). One result of a positive significant association was observed for parental support and general PA (1/7). Evidence on the associations of parental support with leisure-time (1/1) and transport PA (1/1) were unclear. Parental social norm was not associated with change in general (2/2), leisure-time (1/1) and transport PA (1/1). Parental trust in child’s ability to be physically active was also not associated with change in the same domains of general (1/1), leisure-time PA (1/1) and transport PA (1/1). Parents’ enjoyment of PA (1/1), participation in PA (2/2) and parental rules on sedentary behaviour (2/2) were not associated with change in general PA.

Peer influence was investigated in seven papers for six study samples. Friend support was the most examined factor of this group (9/17), followed by friends’ PA and sedentary behaviour (2/17), tease and bullies (2/17), peer-created motivational climate (2/17), number of friends (1/17), and peer acceptance (1/17). No evidence of association between peer influence and change in PA was observed in 15 out of 17 synthesis units. Friend support was not associated with change in general (6/7), leisure-time (1/1), and transport (1/1) PA. One association between friend support and change in general PA was classified as unclear. The association between number of friends (1/1) and general PA was also unclear. Friends’ PA and sedentary behaviour (2/2), tease and bullies (2/2) and peer-created motivational climate (2/2) were not associated with change in general PA, and peer acceptance was not associated with change in school PA (1/1).

Teacher influence was investigated only once in one paper. This study observed no association between teacher support and change in general PA (1/1).

#### Physical environmental factors

Physical environmental factors of nine papers for four different study samples were included in our review (see Tables 5 and 6 and Additional File 6). Transport PA was the domain among which most synthesis units were investigated (44/70), followed by general (34/70), school (14/70), and leisure-time (11/70.). No evidence of association between physical environmental factors and change in PA was observed in the majority of synthesis units (57/70).

The home environment was investigated in one paper. This study observed no associations between active equipment (2/2) and sedentary equipment (2/2) and change in general PA.

The neighbourhood environment was investigated in six papers for three study samples. Subjective characteristics of the neighbourhood environment (e.g. convenience of recreational facilities, residential density, traffic safety) predominated in this group (36/41). Some objective characteristics of the neighbourhood environment (e.g. physical incivilities, territoriality, social spaces, PA facilities) were also investigated (5/41). No evidence of association between the neighbourhood environment and change in PA was observed in the majority of synthesis units (35/41). Subjective characteristics were consistently not associated with change in general (13/15), leisure-time (9/10) and transport PA (9/11). In five out of 36 synthesis units, evidence of association between subjective characteristics of the neighbourhood environment and change in PA was unclear. Objective characteristics of the neighbourhood were not associated with change in general PA in four out of five synthesis units. One positive association was observed between social spaces (e.g. the presence of yards) and change in general PA (1/5).

The school environment was investigated by five papers for four study samples. Factors among this group were extracurricular PA promotion (i.e. active commuting to school, active school yards or playgrounds, health education policy, sports and PA after school, sports and PA during lunch break) (15/25), health education policy (3/25), changing school environment (4/25), difference home-school distance between primary and secondary school (1/25), and supportiveness for walking and cycling (2/25). Papers that reported no association between the school environment and change in PA predominated (19/25). Extracurricular PA promotion was not associated with change in general (4/5), transport (5/5) and school PA (4/5). One result of extracurricular PA promotion and change in general PA was classified unclear (1/5). Furthermore, a positive significant association was observed for extracurricular PA promotion and school PA (1/5). Health education policy was positively associated with change in general PA in one synthesis unit (1/3), while in two other synthesis units of the same study, this factor was not associated with change in general PA (2/3). Changing school environment was not associated with change in general (1/1) and leisure-time PA (1/1), but its association with transport (1/1) and school PA (1/1) was unclear. Association between difference in home-school distance between primary and secondary school and change in transport PA was also unclear (1/1). Supportiveness for walking and cycling was not associated with change in general (1/1) and transport PA (1/1).

#### Additional results

Three qualitative papers on two different study samples investigated factors that were associated with change in PA [48, 49, 52]. These studies identified a number of individual, social and physical environmental factors that changed during the transition from primary to secondary school and that interacted in complex ways to contribute to changes in PA. Examples of individual factors that might be associated with change in PA include changes in motivation, athletic identity, perceived competence, and self-presentation concerns. Examples of social environmental factors possibly associated with change in PA are changes in friends’ involvement in PA, social norms among peers, parents off-loading their PA responsibility to their child and the school, and parents providing their child the independence to walk to and from places without adults. Examples of physical environmental factors possibly associated with change in PA are changes in the physical education environment (e.g. focus on competence and competition), structure of the daily schedule, and academic expectations.

One paper investigated factors associated with change in PA in specific locations [41]. It indicated that PA equipment was positively associated over time with PA at home, and incivilities were negatively associated over time with neighbourhood PA. Parental support, perceived neighbourhood environment, territoriality, social spaces, and outside PA equipment were not associated with either home or neighbourhood PA.

## Discussion

This is the first study to systematically review the current evidence on changes in general and domain-specific PA over the transition period from primary to secondary education. Additionally, individual, social and physical environmental factors associated with these changes were examined. Firstly, most of the evidence on changes in PA and associated factors considered general PA, whereas only a limited number of studies investigated the separate domains of leisure-time, school, and transport. Corroborating previous research, our systematic review showed that general PA is most likely to decline in the transition period from primary to secondary education. Furthermore, studies on school PA mostly reported a decline, but no consistent results were observed for the domains of leisure-time and transport. With respect to the associated factors, studies predominantly included individual factors, and to a lesser degree physical environmental and social environmental factors. Despite the considerable number of factors that were investigated in the different studies, none of the factors were consistently associated with changes in general or domain-specific PA during the school transition period.

Our findings indicate that general PA tends to decline from primary to secondary education. This is in agreement with previous reviews reporting declines in PA during the school transition period [6, 9], and during childhood and adolescence [3, 66]. It supports the notion that the transition to secondary school is a critical period for PA behaviours. Yet, also in line with the literature [6, 9], our findings additionally showed stable and increasing patterns of general PA in some studies suggesting that changes in PA across the school transition period are context-specific, i.e. dependent on PA domain, intensity, and time-period. Importantly, similar to the two previous reviews, the present study replicated the general pattern of results by including twice as many study samples.

Building on the insights of previous reviews, the current review went one step further by also specifically analysing changes in the different domains of PA [6, 9, 10]. Here, we showed that the evidence for the domains of leisure-time and transport was inconclusive. For leisure-time PA, studies reported a decline or an increase for outcomes concerning a combination of PA in organised and non-organised settings, whereas no change was reported for PA in organised settings, and no change or an increase was reported for PA in non-organised settings. For transport PA, studies reported a decline for general active transport, whereas active transport to school was more likely to increase during the school transition period. These findings are similar to the review of Kemp et al. [10], who also reported declining, stable and increasing longitudinal trends for the domains of leisure-time and transport in similar age groups. These results may indicate that, for the domains of leisure-time and transport, change in PA is more specific to the activity itself than to the domain in which the activity occurs. As an example, in the Netherlands, the distance from home to school usually increases with change from primary to secondary education. With most children traveling by bike to primary as well as to secondary schools, this implies that time spent on active travel to school would increase for these children, while there might be less time left to spent on, for example, walking to leisure destinations or other leisure-time activities.

As a further extension of earlier reviews, we also analysed changes in the school domain during the transition from primary to secondary education. Here we found consistent and general evidence which showed that school PA declined. More specifically, the evidence suggests that general school, recess/lunch and extracurricular PA decline over the school transition period while physical education PA increases. These shifts could, in part, be attributed to school environment differences between primary and secondary schools. For example, the SPEEDY study in the UK showed that the duration of school-break times and the provision of extracurricular PA during lunch breaks in secondary schools were significantly less than in primary schools and that these aspects of the school environment were associated with adolescent PA behaviours [67]. In our review, the higher degree of consistency among the results of studies on school PA compared to leisure-time and transport PA may be explained by the structured character of these domains. School is a structured setting with comparable differences for students between primary and secondary schools, whereas environmental differences between primary and secondary schools are more varied with respect to leisure-time and transport settings.

Together, the findings of our review suggest that PA generally declines during the transition period from primary to secondary education. School PA could to a certain extent be considered as a contributor to the general decline. The contributions of the other domains to changes in PA over the school transition period are still not fully understood. The evidence was either equivocal (i.e. leisure-time and transport PA) or not available (home and work PA). Further studies are warranted to explore the changes in domain-specific PA across the transition from primary to secondary education, especially for leisure-time, transport, home and work PA.

In the second part of this review, we examined the influence of individual, social and physical environmental factors on changes in the different domains of PA. A large portion of the previous studies that examined the factors that influence PA in children and adolescents were based on socioecological models. Using these models, a wide range of individual, social and physical environmental factors were identified as contributors to PA. In the majority of studies included in our review, the selection of variables was based on these theoretical considerations and earlier established associations with PA. It is, therefore, surprising that none of the factors in the current review was consistently associated with change in PA across the school transition period. For practical reasons, we chose to summarise the results for each factor in isolation, while socioecological models assume that there is a complex interaction between factors. When considering this interplay between factors, it is understandable that factors do not significantly contribute to changes in PA when examined in isolation. This point of view is strengthened by one qualitative study that indicated that factors are likely to be interdependent with different combinations of factors interacting to shape PA in the separate domains across the transition from primary to secondary education [52]. It should nevertheless be noted that some of the included studies (particularly studies that analysed data from the TRACK sample) did indeed investigate interactions between factors at different socioecological levels (e.g. [33, 41, 44]). Although these studies did not identify many significant interactions, the interactions that were observed were quite impactful and better accounted for change in PA compared to the individual factors. Examples of interactions that were found to be significant are self-efficacy in combination with perceived parent support, self-efficacy in combination with perceptions of the neighbourhood environment and gender in combination with parent’s participation in leisure-time PA.

Together, these findings suggest that, in order to understand and stimulate changes in PA behaviours over the transition period from primary to secondary education, factors should always be considered in combination with other factors. Therefore, for future studies, we consider it as highly important to develop research designs that are suitable for studying the interactions between individual and environmental factors in relation to changes in general and domain-specific PA. Additionally, studies should not only focus on interactions on group levels but also on individual levels, because factors may interact in different ways for different individuals.

In this review, we applied broad inclusion criteria to capture as much of the current evidence as possible. As a consequence, there was a wide variety in how PA outcomes and factors were operationalised and measured, which reflects the field of research on PA. In order to facilitate the evaluation of the evidence, we used multiple decision strategies to summarise the results, such as the categorisation of PA outcomes and factors into smaller groups, and the restriction of results that were reported per study sample. These decisions may have had a negative impact on the depth of our analysis. Nevertheless, given the heterogeneity of the studies, these were necessary steps for the interpretation of the results. Another aspect to be taken into consideration is that we did not include the confounding variables and interactions between factors in the synthesis of our narrative. This means that the results do not reflect the entire complexity of the context in which PA behaviours take place, even though we acknowledge the high importance of including these interactions in future studies. A possible limitation of the results is the fact that most of the studies were conducted in high-income Western settings. In addition, many papers reported results of the same study samples. Both may have affected the generalisability of the findings to lower income regions.

Our study raises a number of opportunities for future research that need to be addressed if we want to have a better understanding of the impact of the school transition period on general and domain-specific PA behaviour. The largest evidence gap identified in this review is the absence of research that investigated changes in the specific domains of PA and its combinations of associated factors. Instead, most studies focused on general PA. Future research is recommended considering the specific domains of PA with both objective and self-report tools, since the current objective measures still do not provide contextual information of PA behaviours (e.g. domain). As an example, objective PA monitors (e.g. accelerometers) can be combined with an electronic diary, in which participants register their activities in real-time [35, 36]. The accelerometer data and the diary output can be translated into contextual information on intensity levels and PA domains, respectively. Additionally, objective GPS loggers [55] could provide information on the physical contexts of PA and self-report questionnaires on the social contexts of PA (e.g. alone, one other, several others, class/team) [58]. Another large evidence gap concerns the investigation of interactions between the different factors. In addition, the investigation of social environmental factors was relatively underrepresented among the included studies. It is well accepted that PA is influenced by individual as well as social and physical environmental characteristics and that these characteristics interact. As the social and physical environments obviously change during the transition period from primary to secondary education (e.g. new school, new friends, new peer groups, new teachers), the lack of emphasis on the environment and the interactions between factors at different socioecological levels needs to be addressed in the future. We suggest that these studies distinguish between non-modifiable and modifiable factors in order to identify the individuals that would benefit most from an intervention as well as the factors that could be targeted with the intervention [68]. Non-modifiable factors that may have an interaction are gender [43], and ethnicity [27]; Modifiable factors that may have an interaction are self-efficacy [40, 43], perceived barriers [40], parental support [32, 40, 43], friend support [40], and objective and subjective characteristics of the home and neighbourhood [32, 40]. Studies should at least include the measurement of these factors, preferably using a combination of child and parent questionnaires, objective tools (e.g. Windshield survey and Physical Activity Recourse Assessment), and qualitative methods such as observations and interviews. A third evidence gap that should be taken into consideration in future research is the lack of studies in non-Western and lower income countries. To better understand the impact of, for example, culture, environment, living-standards and income on PA, studies on changes in PA behaviours during the school transition period in such countries are desirable.

Our study was the first to provide a detailed and systematic understanding of changes in domain-specific PA during the transition period from primary to secondary school, as well as the factors associated with these changes. We thereby extended the work of Chong et al. [9] and Gropper et al. [6]. We included all domains of PA [4] and all its associated factors to provide a holistic picture of PA behaviour in the transition period from primary to secondary school. The current review thereby gives important insights in the state of the knowledge of the PA behaviours in different domains during a critical period of life.

## Conclusions

The current review is a systemic replication of earlier studies showing that general PA is most likely to decline in the transition period from primary to secondary education. Nevertheless, our findings additionally reveal that there is still considerable uncertainty about the exact changes in domain-specific PA behaviours over the transition period from primary to secondary education. Most evidence suggests that PA declines during the school transition period. However, which PA behaviours change, how they change, and why they change remains less clear. This uncertainty is concerning given the societal challenges of physical inactivity (e.g. obesity, less developed motor skills, cardiovascular diseases, mental distress). Our study, therefore, stresses the importance of further research on this topic by providing a broad picture of the research conducted so far on changes in PA across the transition from primary and secondary education. Knowledge of changes in domain-specific PA and its associated factors is required to design promotion strategies to support more active lifestyles during the critical developmental period of transitioning from primary to secondary education.

### Supplementary Information

**Additional file 1.** Search items for PubMed. Search items for Embase. Search items for Web of Science. Search items for PsycINFO. Search items for SPORTDiscus.

**Additional file 2.** Table S1: Decisions for the selection of the PA outcomes and associations.

**Additional file 3.** Table S1: Details of the studies of selected PA outcomes and factors.

**Additional file 4.** Table S1: Assessment of quantitative papers using a modified version of the NHLBI Quality Assessment Tool. Table S2: Assessment of qualitative papers using the JBI Critical Appraisal Checklist for Qualitative Research.

**Additional file 5.** Table S1: Summary of changes in PA synthesis units, including papers. Table S2: Data on changes in PA for each selected paper. Table S3: Summary of reasons to report change in PA as unclear.

**Additional file 6.** Table S1: Summary of associations between demographic factors and change in PA, including papers. Table S2: Summary of associations between factors and change in PA, including papers. Table S3: Data on associations between factors and change in PA for each selected paper. Table S4: Summary of reasons to report associations as unclear.

## Data Availability

The datasets used and/or analysed during the current study are available from the corresponding author on reasonable request.

## Abbreviations

PA: physical activity
PRISMA: Preferred reporting items for systematic Reviews and Meta-analysis guidelines
PROSPERO: International Prospective Register of Systematic Reviews
NHLBI: National Heart, Lung and Blood Institute
JBI: Joanna Briggs Institute
LPA: Light physical activity
MPA: Moderate physical activity
VPA: Vigorous physical activity
MVPA: Moderate-to-vigorous physical activity
PE: Physical education
CADL: Common activities of daily life
TRACK: Transitions and activity changes in kids
