# Supplementary material for: Change in general and domain-specific physical activity during the transition from primary to secondary education: a systematic review
Source: BMC Public Health. 2024 Apr 11;24:1005. doi: 10.1186/s12889-024-18539-1 (PMC11008009; doi:10.1186/s12889-024-18539-1)
Supplement: Supplementary file 1 — Additional file 1. Search items for PubMed. Search items for Embase. Search items for Web of Science. Search items for PsycINFO. Search items for SPORTDiscus. [file 12889_2024_18539_MOESM1_ESM.docx]

## **Search items for PubMed**

### **#1 Physical activity**

Exercise[Mesh] OR Leisure Activities[Mesh] OR Accelerometry[Mesh] OR Fitness Trackers[Mesh] OR exercis*[Tiab] OR leisure activit*[Tiab] OR accelerometr*[Tiab] OR fitness tracker*[Tiab] OR activity monitor*[Tiab] OR physical activit*[Tiab] OR active lifestyl*[Tiab] OR active behavio*[Tiab] OR physically active[Tiab] OR nonorganized PA[Tiab] OR organized PA[Tiab] OR daily step*[Tiab] OR energy expenditure*[Tiab] OR MVPA[Tiab] OR vigorous activit*[Tiab] OR VPA[Tiab] OR moderate activit*[Tiab] OR MPA[Tiab] OR light activit*[Tiab] OR LPA[Tiab] OR intensity activit*[Tiab] OR strenuous activit*[Tiab] OR leisure-time PA[Tiab] OR sport*[Tiab] OR active transport*[Tiab] OR active travel*[Tiab] OR active commut*[Tiab] OR walk*[Tiab] OR cycle[Tiab] OR cycling[Tiab] OR bicycling [tiab] OR active chore*[Tiab] OR household PA[Tiab] OR occupational PA[Tiab] OR job-related PA[Tiab] OR work-related PA[Tiab]

**#2 Transition**

Life Change Events[Mesh] OR life change event*[Tiab] OR life transition*[Tiab] OR life event*[Tiab] OR transition*[Tiab] OR transfer*[Tiab] OR switch*[Tiab] OR trajector*[Tiab] OR change in school* [tiab]

### **#3 Primary or secondary school**

Schools[Mesh] OR primary school*[Tiab] OR primary education*[Tiab] OR elementary school*[Tiab] OR elementary education*[Tiab] OR secondary school*[Tiab] OR secondary education*[Tiab] OR middle school*[Tiab] OR middle education*[Tiab] OR high school*[Tiab] OR highschool* [tiab] OR intermediate school*[Tiab]

### **#4 Participants: children or adolescents**

Child[Mesh:NoExp] OR Adolescent[Mesh] OR Pediatrics[Mesh] OR child*[Tiab] OR schoolchild* [tiab] OR adolescen*[Tiab] OR pediatri* [tiab] OR paediatri* [tiab] OR teen[Tiab] OR teens[Tiab] OR teenager*[Tiab] OR youth*[Tiab] OR boy [Tiab] OR boys[Tiab] OR boyhood [tiab] OR girl [Tiab] OR girls[Tiab] OR girlhood [tiab] OR school-age*[Tiab] OR schoolgoing[tiab] OR school-going[tiab] OR schoolgirl*[tiab] OR schoolboy*[tiab] OR puberty[tiab]

### **#5**

#1 AND #2 AND #3 AND #4

## **Search items for Embase**

### **#1 Physical activity**

exp physical activity/ OR exp Exercise/ OR exp Training/ OR exp Leisure/ OR exp Accelerometry/ OR exp accelerometer/ OR exp activity tracker/ OR exp activity of daily living assessment/ OR (exercis* OR leisure activit* OR accelerometr* OR fitness tracker* OR activity monitor* OR physical activit* OR active lifestyl* OR active behavio* OR physically active OR nonorganized PA OR organized PA OR daily step* OR energy expenditure* OR MVPA OR vigorous activit* OR VPA OR moderate activit* OR MPA OR light activit* OR LPA OR intensity activit* OR strenuous activit* OR leisure-time PA OR sport* OR active transport* OR active travel* OR active commut* OR walk* OR cycle OR cycling OR bicycling OR active chore* OR household PA OR occupational PA OR job-related PA OR work-related PA).ti,ab,kw.

**#2 Transition**

Exp life event/ OR (life change event* OR life transition* OR life event* OR transition* OR transfer* OR switch* OR trajector* OR change in school*).ti,ab,kw.

### **#3 Primary or secondary school**

Exp primary school/ OR exp high school/ OR exp middle school/ OR (primary school* OR primary education* OR elementary school* OR elementary education* OR secondary school* OR secondary education* OR middle school* OR middle education* OR high school* OR highschool* OR intermediate school*).ti,ab,kw.

### **#3 Participants: children or adolescents**

Exp school child/ OR exp adolescent/ OR exp pediatrics/ OR (child* OR schoolchild* OR adolescen* OR pediatri* OR paediatri* OR teen OR teens OR teenager* OR youth* OR boy OR boys OR boyhood OR girl OR girls OR girlhood OR school-age* OR schoolgoing OR school-going OR schoolgirl* OR schoolboy* OR puberty).ti,ab,kw.

### **#5**

#1 AND #2 AND #3 AND #4

## **Search items for Web of Science**

### **#1 Physical activity**

TS=(“exercis*” OR “leisure activit*” OR “accelerometr*” OR “fitness tracker*” OR “activity monitor*” OR “physical activit*” OR “active lifestyl*” OR “active behavio*” OR “physically active” OR “nonorganized PA” OR “organized PA” OR “daily step*” OR “energy expenditure*” OR “MVPA” OR “vigorous activit*” OR “VPA” OR “moderate activit*” OR “MPA” OR “light activit*” OR “LPA” OR “intensity activit*” OR “strenuous activit*” OR “leisure-time PA” OR “sport*” OR “active transport*” OR “active travel*” OR “active commut*” OR “walk*” OR “cycle” OR “cycling” OR “bicycling” OR “active chore*” OR “household PA” OR “occupational PA” OR “job-related PA” OR “work-related PA”)

### **#2 Transition**

TS=(“life change event*” OR “life transition” OR “life event*” OR “transition” OR “transfer*” OR “switch*” OR “trajector*” OR “change in school*”)

**#3 Primary or secondary school**

TS=(“primary school*” OR “primary education*” OR “elementary school*” OR “elementary education*” OR “secondary school*” OR “secondary education*” OR “middle school*” OR “middle education*” OR “high school*” OR “highschool*” OR “intermediate school”)

### **#4 Participants: children or adolescents**

TS=(“child*” OR “schoolchild*” OR “adolescen*” OR “pediatri*” OR “paediatric*” OR “teen” OR “teens” OR “teenager*” OR “youth*” OR “boy” OR “boys” OR “boyhood” OR “girl” OR “girls” OR “girlhood” OR “school-age” OR “schoolgoing” OR “school-going” OR “schoolgirl*” OR “schoolboy*” OR “puberty”)

### **#5**

#1 AND #2 AND #3 AND #4

## **Search items for PsycINFO**

### **#1 Physical activity**

exp Physical Activity/ OR exp Athletic Training/ OR exp Sports/ OR exp Athletic Participation/ OR exp Active Living/ OR exp Daily Activities/ OR exp Recreation/ OR Energy Expenditure/ OR (exercis* OR leisure activit* OR accelerometr* OR fitness tracker* OR activity monitor* OR physical activit* OR active lifestyl* OR active behavio* OR physically active OR nonorganized PA OR organized PA OR daily step* OR energy expenditure* OR MVPA OR vigorous activit* OR VPA OR moderate activit* OR MPA OR light activit* OR LPA OR intensity activit* OR strenuous activit* OR leisure-time PA OR sport* OR active transport* OR active travel* OR active commut* OR walk* OR cycle OR cycling OR bicycling OR active chore* OR household PA OR occupational PA OR job-related PA OR work-related PA).ti,ab,id.

**#2 Transition**

Exp Life Experiences/ OR exp School Transition/ OR (life change event* OR life transition* OR life event* OR transition* OR transfer* OR switch* OR trajector* OR change in school*).ti,ab,id.

### **#3 Primary or secondary school**

Exp Elementary Schools/ OR exp Elementary Education/ OR exp High Schools/ OR exp High School Education/ OR exp Secondary Education/ OR exp Junior High Schools/ OR exp Middle Schools/ OR exp Middle School Education/ OR exp pediatrics/ OR (primary school* OR primary education* OR elementary school* OR elementary education* OR secondary school* OR secondary education* OR middle school* OR middle education* OR high school* OR highschool* OR intermediate school*).ti,ab,id.

### **#4 Participants: children or adolescents**

Exp Elementary School Students/ OR exp Middle School Students/ OR exp Junior High School Students/ OR exp Transfer Students/ OR (child* OR schoolchild* OR adolescen* OR pediatri* OR teen OR teens OR teenager* OR youth* OR boy OR boys OR boyhood OR girl OR girls OR girlhood OR school-age* OR schoolgoing OR school-going OR schoolgirl* OR schoolboy* OR puberty).ti,ab,id.

### **#5**

#1 AND #2 AND #3 AND #4

## **Search items for SPORTDiscus**

### **#1 Physical activity**

DE “physical activity” OR DE “exercise” OR DE “physically active people” OR DE "practice (sports)" OR DE “physical activity measurement” OR DE “sports” OR DE “leisure” OR DE “accelerometers” OR (TI,AB,KW “exercis*” OR “leisure activit*” OR “accelerometr*” OR “fitness tracker*” OR “activity monitor*” OR “physical activit*” OR “active lifestyl*” OR “active behavio*” OR “physically active” OR “nonorganized PA” OR “ ORganized PA” OR “daily step*” OR “energy expenditure*” OR “MVPA” OR “vigorous activit*” OR “VPA” OR “moderate activit*” OR “MPA” OR “light activit*” OR “LPA” OR “intensity activit*” OR “strenuous activit*” OR “leisure-time PA” OR “sport*” OR “active transport*” OR “active travel*” OR “active commut*” OR “walk*” OR “cycle” OR “cycling” OR “bicycling “ OR “active chore*” OR “household PA” OR “occupational PA” OR “job-related PA” OR “work-related PA”)

**#2 Transition**

TI,AB,KW “Life change event*” OR “life transition*” OR “life event*” OR “transition*” OR “transfer*” OR “switch*” OR “trajector*” OR “change in school*

### **#3 Primary or secondary school**

DE “Schools” OR DE “Education” OR (TI,AB,KW “primary school*” OR “primary education*” OR “elementary school*” OR “elementary education*” OR “secondary school*” OR “secondary education*” OR “middle school*” OR “middle education*” OR “high school*” OR “highschool* OR “intermediate school*”)

### **#4 Participants: children or adolescents**

DE “Children” OR DE “School children” OR DE “Boys” OR DE “Girls” OR DE “Teenagers” OR DE “Youth” OR DE “Pediatrics” OR (TI,AB,KW “child*” OR “schoolchild*” OR “adolescen*” OR “pediatri*” OR “paediatri*” OR “teen” OR “teens” OR “teenager*” OR “youth*” OR “boy” OR “boys” OR “boyhood” OR “girl” OR “girls” OR “girlhood” OR “school-age*” OR “schoolgoing” OR “school-going” OR “schoolgirl*” OR “schoolboy*” OR “puberty”)

### **#5**

#1 AND #2 AND #3 AND #4
