# Supplementary material for: Change in general and domain-specific physical activity during the transition from primary to secondary education: a systematic review
Source: BMC Public Health. 2024 Apr 11;24:1005. doi: 10.1186/s12889-024-18539-1 (PMC11008009; doi:10.1186/s12889-024-18539-1)
Supplement: Supplementary file 4 — Additional file 4. Table S1: Assessment of quantitative papers using a modified version of the NHLBI Quality Assessment Tool. Table S2: Assessment of qualitative papers using the JBI Critical Appraisal Checklist for Qualitative Research. [file 12889_2024_18539_MOESM4_ESM.docx]

Table S1. Assessment of quantitative papers using a modified version of the NHLBI Quality Assessment Tool

| **Lead author** | **Year** | **1** | **2** | **3** | **4** | **5** | **6** | **7** | **8** | **9** | **10** | **11** | **12** | **13** | **14** | **15** | **Score** |
| --- | --- | --- | --- | --- | --- | --- | --- | --- | --- | --- | --- | --- | --- | --- | --- | --- | --- |
| Barr-Anderson | 2017 | Y | Y | NR | Y | N | Y | NR (2) | NR | Y | Y | Y | Y | Y | NR | gender, parent education, poverty index, schools | 69% |
| Britton | 2019 | Y | N | NR | Y | N | Y | 6-12 | Y | Y | Y | Y | Y | Y | Y |  | 77% |
| Cardon | 2012 | Y | Y | Y | Y | N | Y | ±12 (4) | Y | Y | Y | N | Y | Y | N |  | 77% |
| Chong | 2022a | Y | Y | Y | Y | N | Y | ±24 | NR | Y | Y | Y | Y | Y | N | gender, main language spoken at home, SES, BMI, pubertal progression, general health status, bullying, change in school environment, season of measurement | 77% |
| Chong | 2022b | Y | Y | N | N | N | NA | 4-16 | NR | NA | N | NA | Y | Y | N | gender, SES, T1 weight status | 40% |
| Clennin | 2019 | Y | Y | Y | Y | Y | Y | NR | NR | Y | Y | Y | Y | Y | Y | age, gender, race, parent education, weight status | 92% |
| Colabianchi | 2016 | Y | Y | NR | Y | N | Y | ±12 | Y | Y | Y | N | Y | Y | Y | gender, race, school district | 77% |
| Colabianchi | 2019 | Y | Y | Y | Y | N | Y | ±12 (2) | NR | Y | Y | Y | Y | Y | NR | race, gender, neighbourhood SES, schools | 77% |
| Coombes | 2014 | Y | Y | Y | N | N | Y | ±12 | Y | Y | Y | Y | Y | Y | N | schools, baseline PA, accelerometer wear time, change in distance to school, daylight | 77% |
| Cooper | 2012 | Y | Y | Y | N | N | Y | ±12 | Y | Y | Y | Y | Y | Y | N | baseline PA, age, BMI, pubertal status, gender, IMD, daylight, schools | 77% |
| De Baere | 2015a | Y | N | NR | NR | N | N | NA | NA | Y | Y | Y | Y | Y | NA |  | 55% |
| De Baere | 2015b | Y | N | NR | NR | N | N | NA | NA | Y | Y | Y | Y | Y | NA | weight status | 55% |
| De Meester | 2014 | Y | Y | Y | Y | N | Y | 24+ | Y | Y | Y | N | Y | Y | N | SES, type of monitor | 77% |
| D'Haese | 2015 | Y | Y | Y | Y | N | Y | 24+ | Y | Y | Y | Y | Y | Y | N | SES, type of monitor | 85% |
| D'Haese | 2016 | Y | Y | Y | Y | N | Y | 24+ | Y | Y | Y | Y | Y | Y | N | SES, type of monitor | 85% |
| Dishman | 2017 | Y | Y | NR | Y | N | Y | NR (2) | NR | Y | Y | Y | Y | Y | Y | gender, race, parental education, poverty level, maturity | 77% |
| Dowda | 2017 | Y | Y | Y | Y | N | Y | NR (2) | NR | Y | Y | Y | Y | Y | N | race, parent education, maturity offset | 77% |
| Dowda | 2020 | Y | Y | NR | Y | N | Y | NR (2) | NR | Y | Y | Y | N | N | N | gender, race, parent education, school | 54% |
| Dowda | 2021 | Y | Y | NR | Y | N | Y | NR | NR | Y | Y | Y | Y | Y | N | gender, race/ethnicity, parent education | 69% |
| Eime | 2016 | Y | Y | N | Y | Y | NA | NR;  ±12 (2) | NR | NA | Y | NA | Y | Y | N |  | 70% |
| Forthofer | 2017 | Y | Y | Y | Y | N | Y | NR | NR | Y | N | Y | Y | Y | N | schools, race, parent education, poverty level | 69% |
| Garcia | 1998 | Y | N | NR | N | N | Y | ±12 | Y | Y | Y | Y | Y | Y | NR |  | 62% |
| Jaakkola | 2019 | Y | Y | Y | N | N | Y | ±12 | NR | Y | Y | Y | Y | Y | NR | pubertal stage, body fat | 69% |
| Jago | 2012 | Y | Y | Y | N | N | Y | ±12 | Y | Y | Y | Y | Y | Y | NR | BMI, IMD, pubertal status, daylight, schools | 77% |
| Johansen | 2023 | Y | Y | Y | Y | Y | NA | ±12 (2) | Y | NA | Y | NA | Y | Y | N |  | 90% |
| Knowles | 2009 | Y | N | NR | Y | N | Y | ±12 | Y | Y | Y | Y | Y | Y | N |  | 69% |
| Lau | 2017 | Y | N | NR | Y | N | Y | ±12 (2) | NR | Y | Y | Y | Y | Y | Y | schools, race, SES | 69% |
| Marks | 2015 | Y | Y | N | Y | N | Y | 5-8 | N | Y | Y | N | Y | Y | Y | accelerometer wear time, baseline PA | 69% |
| Mikalsen | 2020 | Y | Y | Y | Y | N | Y | ±12 (2) | Y | Y | Y | Y | Y | Y | Y | gender | 92% |
| Okazaki | 2022 | Y | Y | NR | N | N | NA | ±12 (4) | Y | NA | Y | NA | Y | Y | Y | gender, weight status, accelerometer wear time | 70% |
| Pate | 1999 | Y | N | Y | Y | N | Y | NR (2) | Y | Y | Y | Y | Y | Y | N |  | 77% |
| Pate | 2019a | Y | Y | NR | Y | N | Y | NR | NR | Y | Y | Y | Y | Y | N | parent education, poverty rate, gender, race, maturation | 69% |
| Remmers | 2020 | Y | Y | N | Y | N | NA | ±12 | Y | NA | Y | NA | Y | Y | N | gender, age, accelerometer wear time, schools, weather | 70% |
| Ridley | 2019 | Y | Y | N | Y | Y | Y | ±12 | Y | Y | Y | Y | Y | Y | N | baseline PA | 85% |
| Rutten | 2014 | N | Y | NR | Y | N | Y | ±24 | Y | Y | Y | Y | Y | Y | N | SES, pubertal development | 69% |
| Saunders | 2018 | Y | Y | Y | Y | N | Y | NR | Y | Y | Y | N | Y | Y | NR | race, parent education | 77% |
| Shin | 2019 | Y | N | NR | Y | N | Y | NR | NR | Y | Y | N | Y | N | NR | gender, SES, peer acceptance | 46% |
| Taylor | 2014 | Y | N | NR | Y | N | Y | 5-9 (2) | N | Y | Y | Y | Y | Y | N | schools | 62% |
| Vanwolleghem | 2016 | Y | Y | Y | Y | N | Y | 24+ | Y | Y | Y | Y | Y | Y | N | baseline PA | 85% |
| Zhu | 2017 | Y | Y | Y | Y | N | N | NA | NA | Y | N | N | Y | N | NA | grade, gender, BMI | 55% |

Items: (1) Was the research question or objective in this paper clearly stated?; (2) Was the study population clearly specified and defined?; (3) Was the participation rate of eligible persons at least 50%?; (4) Were all the subjects selected or recruited from the same or similar populations (including the same time period)? Were inclusion and exclusion criteria for being in the study prespecified and applied uniformly to all participants?; (5) Was a sample size justification, power description, or variance and effect estimates provided?; (6) For the analyses in this paper, were the independent variable(s) of interest measured prior to the dependent variable(s) being measured? (7) What was the timeframe between the assessment of independent variable(s) and dependent variable(s)? – Number of follow-ups is between brackets; (8) Was the timeframe sufficient so that one could reasonably expect to see an association between the independent variable and dependent variable? (9) For independent variables that can vary in amount or level, did the study examine different levels of the independent variable as related to the dependent variable (e.g., categories of independent variable, or independent variable as continuous variable)?; (10) Were the independent variables clearly defined?; (11) Were the independent variables valid and reliable?; (12) Were the dependent variables clearly defined?; (13) Were the dependent variables valid and reliable?; (14) Was loss to follow-up after baseline 20% or less?; (15) Which key potential confounding variables were measured and adjusted statistically for their impact on the relationship between independent and dependent variable(s)?

Table S2. Assessment of qualitative papers using the JBI Critical Appraisal Checklist for Qualitative Research

| **Lead author** | **Year** | **1** | **2** | **3** | **4** | **5** | **6** | **7** | **8** | **9** | **10** | **Score** |
| --- | --- | --- | --- | --- | --- | --- | --- | --- | --- | --- | --- | --- |
| Knowles | 2011 | Y | Y | Y | Y | N | N | N | N | Y | Y | 60% |
| Knowles | 2014 | Y | Y | Y | Y | Y | Y | Y | Y | Y | Y | 100% |
| McGaughey | 2020 | Y | N | Y | **N** | N | N | N | Y | Y | Y | 50% |

Items: (1) Is there congruity between the stated philosophical perspective and the research methodology?; (2) Is there congruity between the research methodology and the research question or objective?; (3) Is there congruity between the research methodology and the methods used to collect data?; (4) Is there congruity between the research methodology and the representation and analysis of data?; (5) Is there congruity between the research methodology and the interpretation of results?; (6) Is there a statement locating the researcher culturally or theoretically?; (7) Is the influence of the researcher on the research, and vice-versa, addressed?; (8) Are participants, and their voices, adequately represented?; (9) Is the research ethical according to current criteria or, for recent studies, and is there evidence of ethical approval by an appropriate body?; (10) Do the conclusions drawn in the research report flow from the analysis, or interpretation, of the data?
