# Supplementary material for: Change in general and domain-specific physical activity during the transition from primary to secondary education: a systematic review
Source: BMC Public Health. 2024 Apr 11;24:1005. doi: 10.1186/s12889-024-18539-1 (PMC11008009; doi:10.1186/s12889-024-18539-1)
Supplement: Supplementary file 5 — Additional file 5. Table S1: Summary of changes in PA synthesis units, including papers. Table S2: Data on changes in PA for each selected paper. Table S3: Summary of reasons to report change in PA as unclear. [file 12889_2024_18539_MOESM5_ESM.docx]

Table S1. Summary of changes in PA synthesis units, including papers

| **PA domain** | **PA synthesis units** | **-** | **=** | **+** | **unclear** |
| --- | --- | --- | --- | --- | --- |
| **General** | All intensities / Total PA (objective) | 2 | 2 | 0 | 0 |
|  |  | Dishman (2017) Rutten (2014) | Coombes (2014) De Baere (2015b) |  |  |
|  | All intensities / Total PA (subjective) | 4 | 3 | 1 | 1 |
|  |  | De Meester (2014) Knowles (2009) Ridley (2019) Taylor (2014) | Dowda (2021) Garcia (1998) Zhu (2017) | Pate (1999) | Chong (2022a) |
|  | MVPA (objective) | 6 | 1 | 2 | 1 |
|  |  | Britton (2019) Chong (2022b) Dowda (2017) Marks (2015) Mikalsen (2020) Okazaki (2022) | Remmers (2020) | De Meester (2014) Cooper (2012) | Jaakkola (2019) |
|  | MVPA (subjective) | 1 | 0 | 0 | 0 |
|  |  | Pate (1999) |  |  |  |
|  | LPA (objective) | 4 | 1 | 0 | 0 |
|  |  | De Baere (2015b) Chong (2022b) Marks (2015) Okazaki (2022) | Remmers (2020) |  |  |
|  | MPA (objective) | 0 | 0 | 1 | 0 |
|  |  |  |  | De Baere (2015b) |  |
|  | VPA (objective) | 0 | 0 | 1 | 0 |
|  |  |  |  | De Baere (2015b) |  |
|  | VPA (subjective) | 0 | 1 | 0 | 0 |
|  |  |  | Pate (1999) |  |  |
|  | Number of steps | 1 | 2 | 0 | 0 |
|  |  | De Baere (2015b) | De Meester (2014) Rutten (2014) |  |  |
| **School** | General school (objective) | 3 | 0 | 0 | 0 |
|  |  | De Baere (2015b) Johansen (2023) Lau (2017) |  |  |  |
|  | General school (subjective) | 1 | 0 | 0 | 0 |
|  |  | Rutten (2014) |  |  |  |
|  | Recess/lunch (objective) | 0 | 0 | 0 | 1 |
|  |  |  |  |  | De Baere (2015a) |
|  | Recess/lunch (subjective) | 2 | 0 | 0 | 0 |
|  |  | Marks (2015) Ridley (2019) |  |  |  |
|  | Physical education (subjective) | 0 | 1 | 2 | 1 |
|  |  |  | Ridley (2019) | Eime (2016) Shin (2019) | Marks (2015) |
|  | Extracurricular PA (subjective) | 2 | 1 | 0 | 0 |
|  |  | De Meester (2014) Eime (2016) | Chong (2022b) |  |  |
| **Leisure-time** | Organized and non-organized (objective) | 0 | 0 | 1 | 0 |
|  |  |  |  | De Baere (2015b) |  |
|  | Organized and non-organized (subjective) | 2 | 0 | 0 | 2 |
|  |  | D'Haese (2015) Rutten (2014) |  |  | Marks (2015) Ridley (2019) |
|  | Organized (subjective) | 0 | 2 | 0 | 0 |
|  |  |  | Chong (2022b) Eime (2016) |  |  |
|  | Non-organized PA (objective) | 0 | 1 | 0 | 0 |
|  |  |  | De Baere (2015b) |  |  |
|  | Non-organized PA (subjective) | 0 | 0 | 1 | 0 |
|  |  |  |  | Eime (2016) |  |
| **Transport** | General active transport (objective) | 1 | 0 | 0 | 1 |
|  |  | De Baere (2015b) |  |  | Remmers (2020) |
|  | Active transport to school (subjective) | 1 | 0 | 2 | 1 |
|  |  | Cooper (2012) |  | De Meester (2014) Cardon (2012) | Marks (2015) |
|  | Active transport during leisure (subjective) | 0 | 0 | 0 | 1 |
|  |  |  |  |  | D'Haese (2015) |
| **Home** | Common activities of daily life (objective) | 0 | 1 | 0 | 0 |
|  |  |  | De Baere (2015b) |  |  |

Change is reported as negative (-), no change (=), positive (+) or unclear.

Table S2. Data on changes in PA for each selected paper

|  |  |  |  |  |  | **PA change** | | | |
| --- | --- | --- | --- | --- | --- | --- | --- | --- | --- |
| **Lead author** | **Year** | **PA assessment tools** | **PA outcomes** | **PA synthesis units** | **PA domain** | 0-12 mth post-transition | 12-24 mth post-transition | 0-24 mth post-transition | Reported |
| Britton | 2019 | Accelerometry (9 days) | MVPA (min/day) | MVPA (objective) | General | - |  |  | - |
| Cardon | 2012 | Q (FPAQ) | Bicycling to school (min/day during roundtrip) | Active transport to school (subjective) | Transport | + | + |  | + |
| Chong | 2022a | Q | Schoolday PA (min/day) | All intensities / Total PA (subjective) | General |  |  | - | unclear |
|  |  |  | Nonschool day PA (min/day) |  | General |  |  | = |  |
| Chong | 2022b | Accelerometry (6 days) | LPA (min/day) | LPA (objective) | General | - |  |  | - |
|  |  |  | MVPA (min/day) | MVPA (objective) | General | - |  |  | - |
|  |  | Q (adapted from Eime, 2016) | Competitive team activities in school (yes/no) | Extracurricular PA (subjective) | School | = |  |  | = |
|  |  |  | Competitive individual activities in school (yes/no) |  | School | = |  |  |  |
|  |  |  | Competitive team activities out of school (yes/no) | Organized (subjective) | Leisure-time | = |  |  | = |
|  |  |  | Competitive individual activities out of school (yes/no) |  | Leisure-time | = |  |  |  |
|  |  |  | Organized non-competitive activities (yes/no) |  | Leisure-time | = |  |  |  |
| Coombes | 2014 | Accelerometry (7 days) | PA level (daily counts/min) | All intensities / Total PA (objective) | General | = |  |  | = |
| Cooper | 2012 | Accelerometry (7 days) | Weekday MVPA (min/day) | MVPA (objective) | General | + |  |  | + |
|  |  | Q | Travel mode to school (walk/car/bus) | Active transport to school (subjective) | Transport | - |  |  | - |
| De Baere | 2015a | Triaxial accelerometry with physiological measures and electronic diary (7 days) | Proportion LPA during recess time (%) | Recess/lunch (objective) | School |  |  | - | unclear |
|  |  |  | Proportion MPA during recess time (%) |  | School |  |  | = |  |
|  |  |  | Proportion VPA during recess time (%) |  | School |  |  | = |  |
| De Baere | 2015b | Triaxial accelerometry with physiological measures and electronic diary (7 days) | PA Level (METswm) | All intensities / Total PA (objective) | General |  |  | = | = |
|  |  |  | Steps (n/day) | Number of steps | General |  |  | - | - |
|  |  |  | LPA (min/day) | LPA (objective) | General |  |  | - | - |
|  |  |  | MPA (min/day) | MPA (objective) | General |  |  | + | + |
|  |  |  | VPA (min/day) | VPA (objective) | General |  |  | + | + |
|  |  |  | School (min/day) | School (objective) | School |  |  | - | - |
|  |  |  | Sport (min/day) | Organized and non-organized (objective) | Leisure-time |  |  | + | + |
|  |  |  | Active leisure (min/day) | Non-organized (objective) | Leisure-time |  |  | = | = |
|  |  |  | CADL (min/day) | Common activities of daily life (objective) | Home |  |  | = | = |
|  |  |  | Active travel (min/day) | Active transport (objective) | Transport |  |  | - | - |
| De Meester | 2014 | Q (FPAQ) | Active transport to school (mean min/day) | Active transport to school (subjective) | Transport |  |  | + | + |
|  |  |  | Extracurricular PA (min/day) | Extracurricular PA (subjective) | School |  |  | - | - |
|  |  |  | Total PA level (mean min/day) | All intensities / Total PA (subjective) | General |  |  | - | - |
|  |  | Accelerometry and pedometry (7 days) | Pedometer/accelerometer weekday steps (mean n/day) | Number of steps | General |  |  | = | = |
|  |  |  | Accelerometer weekday MVPA (mean min/day) | MVPA (objective) | General |  |  | + | + |
| D'Haese | 2015 | Q (FPAQ) | Sports during leisure (min/day) | Organized and non-organized (subjective) | Leisure-time |  |  | - | - |
|  |  |  | Walking for transport during leisure (min/day) | Active transport during leisure (subjective) | Transport |  |  | = G / - B | unclear |
|  |  |  | Cycling for transport during leisure (min/day) |  | Transport |  |  | = |  |
| Dishman | 2017 | Accelerometry (7 days) | Total PA (min/hr) | All intensities / Total PA (objective) | General | - | - |  | - |
| Dowda | 2017 | Accelerometry (7 days) | MVPA (min/hr) | MVPA (objective) | General |  |  | - | - |
| Dowda | 2021 | Q | PA level (child-reported) | All intensities / Total PA (subjective) | General |  |  | = | = |
|  |  |  | PA level (parent-reported) |  | General |  |  | = |  |
| Eime | 2016 | Q (n.r.) | School physical education classes (yes/no) | Physical education (subjective) | School |  |  | + | + |
|  |  |  | Competitive team activities in school (yes/no) | Extracurricular PA (subjective) | School |  |  | - | - |
|  |  |  | Competitive individual activities in school (yes/no) |  | School |  |  | - |  |
|  |  |  | Competitive team activities out of school (yes/no) | Organized (subjective) | Leisure-time |  |  | = | = |
|  |  |  | Competitive individual activities out of school (yes/no) |  | Leisure-time |  |  | = |  |
|  |  |  | Organized non-competitive activities (yes/no) |  | Leisure-time |  |  | = |  |
|  |  |  | Non-organized activities (yes/no) | Non-organized (subjective) | Leisure-time |  |  | + | + |
| Garcia | 1998 | Q (CAAL, 7 days) | PA level (index of total effort) | All intensities / Total PA (subjective) | General | = |  |  | = |
| Jaakkola | 2019 | Accelerometry (7 days) | MVPA (min/day) | MVPA (objective) | General | - G / = B |  |  | unclear |
| Johansen | 2023 | Accelerometry (7 days) | MVPA during schooltime (min/wk) | School (objective) | School |  |  | - | - |
| Knowles | 2009 | Q (PAQ-C) | PA level (range 1-5) | All intensities / Total PA (subjective) | General | - |  |  | - |
| Lau | 2017 | Accelerometry (7 days) | School Total PA (min/hr) | School (objective) | School |  |  | - | - |
| Marks | 2015 | Q (items from PAQ-C) | Being very active in physical education class (frequency) | Physical education (subjective) | School | sig. |  |  | unclear |
|  |  |  | Do most of the time at recess | Recess/lunch (subjective) | School | - |  |  | - |
|  |  |  | Do most of the time at lunch |  | School | - |  |  |  |
|  |  | Q (items from ABAKQ) | Walking to school (times/wk) | Active transport to school (subjective) | Transport | = |  |  | unclear |
|  |  |  | Cycle/scoot to school (times/wk) |  | Transport | - |  |  |  |
|  |  | Q (combination of items PAQ-C & CLASS) | After-school being very active (min/day) | Organized and non-organized (subjective) | Leisure-time | + |  |  | unclear |
|  |  |  | Weekend being very active (min/day) |  | Leisure-time | = |  |  |  |
|  |  | Accelerometry (7 days) | MVPA (min/day) | MVPA (objective) | General | - |  |  | - |
|  |  |  | LPA (min/day) | LPA (objective) | General | - |  |  | - |
| Mikalsen | 2020 | Accelerometry (7 days) | MVPA (min/day) | MVPA (objective) | General |  |  | - | - |
| Okazaki | 2022 | Accelerometry (7 days) | MVPA (min/day) | MVPA (objective) | General |  |  | - | - |
|  |  |  | LPA (min/day) | LPA (objective) | General |  |  | - | - |
| Pate | 1999 | Q (PDPAR, 3 days) | VPA (# of 30-min blocks) | VPA (subjective) | General | + | - | = | = |
|  |  |  | MVPA (# of 30-min blocks) | MVPA (subjective) | General | + | - | - | - |
|  |  |  | After-school Kcals | All intensities / Total PA (subjective) | General | + | = | + | + |
| Remmers | 2020 | Accelerometry and GPS log (7 days) | Weekend days LPA (min/day) | LPA (objective) | General | = |  |  | = |
|  |  |  | Weekdays LPA (min/day) |  | General | = |  |  |  |
|  |  |  | Weekend days MVPA (min/day) | MVPA (objective) | General | = |  |  | = |
|  |  |  | Weekdays MVPA (min/day) |  | General | = |  |  |  |
|  |  |  | Weekend days Active transport LPA (min/day) | Active transport (objective) | Transport | - |  |  | unclear |
|  |  |  | Weekend days Active transport MVPA (min/day) |  | Transport | = |  |  |  |
|  |  |  | Weekdays before school Active transport LPA (min/day) |  | Transport | + |  |  |  |
|  |  |  | Weekdays before school Active transport MVPA (min/day) |  | Transport | = |  |  |  |
|  |  |  | Weekdays during school Active transport LPA (min/day) |  | Transport | = |  |  |  |
|  |  |  | Weekdays during school Active transport MVPA (min/day) |  | Transport | = |  |  |  |
|  |  |  | Weekdays after school Active transport LPA (min/day) |  | Transport | = |  |  |  |
|  |  |  | Weekdays after school Active transport MVPA (min/day) |  | Transport | = |  |  |  |
| Ridley | 2019 | Q (PAQ-C) | PA level (range 1-5) | All intensities / Total PA (subjective) | General | - |  |  | - |
|  |  |  | After school PA level (range 1-5) | Organized and non-organized (subjective) | Leisure-time |  |  | = | unclear |
|  |  |  | Evenings PA level (range 1-5) |  | Leisure-time |  |  | = |  |
|  |  |  | Weekends PA level (range 1-5) |  | Leisure-time |  |  | - |  |
|  |  |  | Physical education PA level (range 1-5) | Physical education (subjective) | School | = |  |  | = |
|  |  |  | Lunch/recess PA level (range 1-5) | Recess/lunch (subjective) | School | - |  |  | - |
| Rutten | 2014 | Pedometry (7 days) | Steps (n/day) | Number of steps | General |  |  | = | = |
|  |  | Q (PAQ-C) | PA level (range 1-5) | All intensities / Total PA (objective) | General |  |  | - | - |
|  |  |  | PA level at school (range 1-5) | School (subjective) | School |  |  | - | - |
|  |  |  | PA level during leisure time (range 1-5) | Organized and non-organized (subjective) | Leisure-time |  |  | - | - |
| Shin | 2019 | Q | Perceived physical education activity (range 1-5) | Physical education (subjective) | School | + |  |  | + |
| Taylor | 2014 | Q (PAQ-C) | PA level (range 1-5) | All intensities / Total PA (subjective) | General | - | = | - | - |
| Zhu | 2017 | Q (items from Active Healthy Global Alliance study) | PA for a total of at least 60 minutes per day (d/wk) | All intensities / Total PA (subjective) | General | = | | | = |

Q = questionnaire; FPAQ = Flemish Physical Activity Questionnaire; n.r. = not reported; CAAL = Child/Adolescent Activity Log; PAQ-C = Physical Activity Questionnaire for older Children; ABAKQ = Adolecent Behaviours, Attitudes, and Knowledge Questionnaire; CLASS = Children’s Leisure Activities Study Survey; PDPAR = Previous Day Physical Activity Recall; GPS = Global Positioning System; PA = physical activity; LPA = light physical activity; MPA = moderate physical activity; VPA = vigorous physical activity; MVPA = moderate-to-vigorous physical activity; CADL = common activities of daily life; G = girls; B = boys. Change is reported as negative (-), no change (=), positive (+) or unclear.

Table S3. Summary of reasons to report change in PA as unclear

|  |  | **Domain** | | | | **Reason for unclear** | | | |
| --- | --- | --- | --- | --- | --- | --- | --- | --- | --- |
| **Sample** | **Author (year)** | **General** | **Leisure-time** | **Transport** | **School** | **1** | **2** | **3** | **4** |
|  | Chong (2022a) | 1 |  |  |  | 1 |  |  |  |
| De Baere (2015a) | De Baere (2015a) |  |  |  | 1 | 1 |  |  |  |
| De Meester (2014) | D’Haese (2015) |  |  | 1 |  |  |  | 1 |  |
|  | Jaakkola (2019) | 1 |  |  |  |  | 1 |  |  |
|  | Marks (2015) |  | 1 | 1 | 1 | 2 |  |  | 1 |
| PHASE | Remmers (2020) |  |  | 1 |  | 1 |  |  |  |
|  | Ridley (2019) |  | 1 |  |  | 1 |  |  |  |

PHASE = Physical Activity in Public Space Environments; Reasons for unclear: 1 = Differences in results between PA outcomes; 2 = Differences in results between subgroups (e.g., gender); 3 = Combination of differences in results between PA outcomes and subgroups (e.g., gender); 4 = Direction of change unknown.
