# Supplementary material for: Change in general and domain-specific physical activity during the transition from primary to secondary education: a systematic review
Source: BMC Public Health. 2024 Apr 11;24:1005. doi: 10.1186/s12889-024-18539-1 (PMC11008009; doi:10.1186/s12889-024-18539-1)
Supplement: Supplementary file 6 — Additional file 6. Table S1: Summary of associations between demographic factors and change in PA, including papers. Table S2: Summary of associations between factors and change in PA, including papers. Table S3: Data on associations between factors and change in PA for each selected paper. Table S4: Summary of reasons to report associations as unclear. [file 12889_2024_18539_MOESM6_ESM.docx]

Table S1. Summary of associations between demographic factors and change in PA, including papers

|  |  | **General** | | | **Leisure-time** | | | **Transport** | | | | **School** | | | **Home** | | |
| --- | --- | --- | --- | --- | --- | --- | --- | --- | --- | --- | --- | --- | --- | --- | --- | --- | --- |
| **Level of influence** | **Group of factors** | **n.s.** | **sig.** | **unclear** | **n.s.** | **sig.** | **unclear** | **n.s.** | **sig.** | **unclear** | **n.s.** | | **sig.** | **unclear** | **n.s.** | **sig.** | **unclear** |
| **Individual** | Demographic | 7 | 0 | 0 | 2 | 0 | 0 | 3 | 0 | 0 | 4 | | 0 | 0 | 1 | 0 | 0 |
|  |  | Barr-Anderson (2017) Britton (2019) De Baere (2015b) De Meester (2014) Pate (2019a) Rutten (2014) |  |  | De Baere (2015b) Rutten (2014) |  |  | De Baere (2015b) De Meester (2014) Vanwolleghem (2016) |  |  | De Baere (2015b) De Meester (2014) Rutten (2014) Shin (2019) | |  |  | De Baere (2015b) |  |  |

Association is reported as not significant (n.s.) or significant (sig.).

Table S2. Summary of associations between factors and change in PA, including papers

|  |  | **General** | | | | **Leisure-time** | | | | **Transport** | | | | **School** | | | |
| --- | --- | --- | --- | --- | --- | --- | --- | --- | --- | --- | --- | --- | --- | --- | --- | --- | --- |
| **Level of influence** | **Group of factors** | **n.s.** | **+** | **-** | **unclear** | **n.s.** | **+** | **-** | **unclear** | **n.s.** | **+** | **-** | **unclear** | **n.s.** | **+** | **-** | **unclear** |
| **Individual** | Physical characteristics | 5 | 0 | 0 | 0 | 0 | 0 | 1 | 0 | 0 | 0 | 0 | 0 | 1 | 0 | 0 | 0 |
|  |  | Rutten (2014) Knowles (2009) |  |  |  |  |  | Rutten (2014) |  |  |  |  |  | Rutten (2014) |  |  |  |
|  | Motivation and goals | 10 | 2 | 0 | 2 | 1 | 0 | 0 | 0 | 1 | 0 | 0 | 0 | 0 | 0 | 0 | 0 |
|  |  | Barr-Anderson (2017) Pate (2019a) Taylor (2014) | Mikalsen (2020) Ridley (2019) |  | Barr-Anderson (2017) D'Haese (2016) | D'Haese (2016) |  |  |  | D'Haese (2016) |  |  |  |  |  |  |  |
|  | Self-concept | 4 | 1 | 0 | 0 | 0 | 0 | 0 | 0 | 0 | 0 | 0 | 0 | 1 | 0 | 0 | 0 |
|  |  | Knowles (2009) Pate (2019a) | Knowles (2009) |  |  |  |  |  |  |  |  |  |  | Shin (2019) |  |  |  |
|  | Beliefs about capabilities | 6 | 1 | 0 | 0 | 0 | 0 | 0 | 1 | 1 | 0 | 0 | 0 | 0 | 0 | 0 | 0 |
|  |  | Colabianchi (2019) D'Haese (2016) Knowles (2009) Pate (2019a) Ridley (2019) | Mikalsen (2020) |  |  |  |  |  |  | D'Haese (2016) |  |  |  |  |  |  |  |
|  | Beliefs about consequences | 12 | 0 | 0 | 2 | 10 | 0 | 0 | 1 | 8 | 0 | 0 | 4 | 0 | 0 | 0 | 0 |
|  |  | D'Haese (2016) Pate (2019a) Ridley (2019) |  |  | D'Haese (2016) | D'Haese (2016) |  |  | D'Haese (2016) | D'Haese (2016) Vanwolleghem (2016) |  |  | D'Haese (2016) |  |  |  |  |
| **Social environmental** | Parental influence | 14 | 1 | 0 | 0 | 2 | 0 | 0 | 1 | 2 | 0 | 0 | 1 | 0 | 0 | 0 | 0 |
|  |  | D'Haese (2016) Mikalsen (2020) Pate (2019a) Ridley (2019) | Pate (2019a) |  |  | D'Haese (2016) |  |  |  | D'Haese (2016) |  |  | D'Haese (2016) |  |  |  |  |
|  | Peer influence | 12 | 0 | 0 | 2 | 1 | 0 | 0 | 0 | 1 | 0 | 0 | 0 | 1 | 0 | 0 | 0 |
|  |  | Barr-Anderson (2017) D'Haese (2016) Jago (2012) Pate (2019a) Ridley (2019) Taylor (2014) |  |  | Jago (2012) | D'Haese (2016) |  |  |  | D'Haese (2016) |  |  |  | Shin (2019) |  |  |  |
|  | Teacher influence | 1 | 0 | 0 | 0 | 0 | 0 | 0 | 0 | 0 | 0 | 0 | 0 | 0 | 0 | 0 | 0 |
|  |  | Taylor (2014) |  |  |  |  |  |  |  |  |  |  |  |  |  |  |  |
| **Physical environmental** | Home equipment | 4 | 0 | 0 | 0 | 0 | 0 | 0 | 0 | 0 | 0 | 0 | 0 | 0 | 0 | 0 | 0 |
|  |  | Pate (2019a) |  |  |  |  |  |  |  |  |  |  |  |  |  |  |  |
|  | Neighborhood environment | 17 | 1 | 0 | 2 | 9 | 0 | 0 | 1 | 9 | 0 | 0 | 2 | 0 | 0 | 0 | 0 |
|  |  | Clennin (2019) Coombes (2014) D'Haese (2015) Forthofer (2017) Pate (2019a) | Pate (2019a) |  | Barr-Anderson (2017) D'Haese (2015) | D'Haese (2015) |  |  | D'Haese (2015) | Coombes (2014) D'Haese (2015) |  |  | D'Haese (2015) |  |  |  |  |
|  | School environment | 8 | 0 | 1 | 1 | 1 | 0 | 0 | 0 | 6 | 0 | 0 | 2 | 4 | 1 | 0 | 1 |
|  |  | Coombes (2014) De Meester (2014) Marks (2015) Pate (2019a) |  | Pate (2019a) | De Meester (2014) | Marks (2015) |  |  |  | Coombes (2014) De Meester (2014) |  |  | Marks (2015) Vanwolleghem (2016) | De Meester (2014) | De Meester (2014) |  | Marks (2015) |

Association is reported as not significant (n.s.), positive/protective against declines (+), negative/contributing to declines (-) or unclear.

Table S3. Results on associations between factors and change in PA for each selected paper

| **PA domain** | **Socioecological level** | **Group of factors** | **Factor** | **Lead author** | **Year** | **PA Outcome** | **Significance** | **Reported** |
| --- | --- | --- | --- | --- | --- | --- | --- | --- |
| General | Individual | Demographic | Gender | Britton | 2019 | MVPA (min/day) | n.s. | n.s. |
|  |  |  | Gender | De Baere | 2015b | PA Level (METswm) | n.s. | n.s. |
|  |  |  |  |  |  | Steps (n/day) | n.s. |  |
|  |  |  | Gender (C) | De Meester | 2014 | Total PA level (mean min/day) | n.s. | n.s. |
|  |  |  |  |  |  | Pedometer/accelerometer weekday steps (mean n/day) | n.s. |  |
|  |  |  |  |  |  | Accelerometer weekday MVPA (mean min/day) | n.s. |  |
|  |  |  | Gender | Rutten | 2014 | Steps (n/day) | n.s. | n.s. |
|  |  |  |  |  |  | PA level (range 1-5) | n.s. |  |
|  |  |  | Race (C) | Barr-Anderson | 2017 | Total PA (min/hr) | n.s. | n.s. |
|  |  |  | Socioeconomic status | Barr-Anderson | 2017 | Total PA (min/hr) | n.s. | n.s. |
|  |  |  | Number of adults in the home (P) | Pate | 2019a | PA (min/hr) | n.s. | n.s. |
|  |  | Physical characteristics | Body mass | Knowles | 2009 | PA level (range 1-5) | n.s. | n.s. |
|  |  |  | Skinfolds | Knowles | 2009 | PA level (range 1-5) | n.s. | n.s. |
|  |  |  | Waist circumference | Knowles | 2009 | PA level (range 1-5) | n.s. | n.s. |
|  |  |  | BMI: Overweight | Rutten | 2014 | Steps (n/day) | n.s. | n.s. |
|  |  |  |  |  |  | PA level (range 1-5) | n.s. |  |
|  |  |  | Maturation (C) | Knowles | 2009 | PA level (range 1-5) | n.s. | n.s. |
|  |  | Motivation and goals | Enjoyment of PA (C)(P) | Barr-Anderson | 2017 | Total PA (min/hr) | n.s. Black (C)(P) / - White (C) / n.s. White (P) / + Hispanic (C) / n.s. Hispanic (P) | unclear |
|  |  |  | Enjoyment of physical education (P) | Barr-Anderson | 2017 | Total PA (min/hr) | n.s. | n.s. |
|  |  |  | Child's attitude toward PA (C)(P) | D'Haese | 2016 | Steps (n/day) | + G (C) / n.s. G (P) / n.s. B (C)(P) | unclear |
|  |  |  | Like PA | Ridley | 2019 | PA level (range 1-5) | + | + |
|  |  |  | Eagerness for PA | Mikalsen | 2020 | MVPA (min/day) | + | + |
|  |  |  | Motives for PA: Enjoyment motivation (C) | Pate | 2019a | PA (min/hr) | n.s. | n.s. |
|  |  |  | Motives for PA: Appearance motivation (C) | Pate | 2019a | PA (min/hr) | n.s. | n.s. |
|  |  |  | Motives for PA: Competence motivation (C) | Pate | 2019a | PA (min/hr) | n.s. | n.s. |
|  |  |  | Motives for PA: Fitness motivation (C) | Pate | 2019a | PA (min/hr) | n.s. | n.s. |
|  |  |  | Motives for PA: Social motivation (C) | Pate | 2019a | PA (min/hr) | n.s. | n.s. |
|  |  |  | Motivational regulations: external motivation | Taylor | 2014 | PA level (range 1-5) | n.s. | n.s. |
|  |  |  | Motivational regulations: identified regulation | Taylor | 2014 | PA level (range 1-5) | n.s. | n.s. |
|  |  |  | Motivational regulations: intrinsic motivation |  |  | PA level (range 1-5) |  |  |
|  |  |  | Motivational regulations: introjected regulation | Taylor | 2014 | PA level (range 1-5) | n.s. | n.s. |
|  |  |  | Motivational regulations: amotivation | Taylor | 2014 | PA level (range 1-5) | n.s. | n.s. |
|  |  | Self-concept | Body attractiveness | Knowles | 2009 | PA level (range 1-5) | n.s. | n.s. |
|  |  |  | Muscular strength and development | Knowles | 2009 | PA level (range 1-5) | n.s. | n.s. |
|  |  |  | Overall physical Self-worth | Knowles | 2009 | PA level (range 1-5) | n.s. | n.s. |
|  |  |  | Physical condition | Knowles | 2009 | PA level (range 1-5) | + | + |
|  |  |  | Self-schema (C) | Pate | 2019a | PA (min/hr) | n.s. | n.s. |
|  |  | Beliefs about capabilities | Self-efficacy | D'Haese | 2016 | Steps (n/day) | n.s. | n.s. |
|  |  |  | Self-efficacy (C) | Pate | 2019a | PA (min/hr) | n.s. | n.s. |
|  |  |  | Barriers self-efficacy | Ridley | 2019 | PA level (range 1-5) | n.s. | n.s. |
|  |  |  | Perception of skill (C) | Colabianchi | 2019 | Total PA (min/hr) | n.s. | n.s. |
|  |  |  | Sport Competence | Knowles | 2009 | PA level (range 1-5) | n.s. | n.s. |
|  |  |  | Good at PA | Ridley | 2019 | PA level (range 1-5) | n.s. | n.s. |
|  |  |  | Perceived athletic competence | Mikalsen | 2020 | MVPA (min/day) | + | + |
|  |  | Beliefs about consequences | Perceived benefit of being better than others (C)(P) | D'Haese | 2016 | Steps (n/day) | n.s. | n.s. |
|  |  |  | Perceived benefit of fun (C)(P) | D'Haese | 2016 | Steps (n/day) | n.s. | n.s. |
|  |  |  | Perceived benefit of health (C)(P) | D'Haese | 2016 | Steps (n/day) | n.s. | n.s. |
|  |  |  | Perceived benefit of meeting (new) friends (C)(P) | D'Haese | 2016 | Steps (n/day) | n.s. | n.s. |
|  |  |  | Perceived benefit of not feeling bored (C)(P) | D'Haese | 2016 | Steps (n/day) | n.s. | n.s. |
|  |  |  | Perceived benefit of weight loss (C)(P) | D'Haese | 2016 | Steps (n/day) | n.s. | n.s. |
|  |  |  | Perceived outcomes of regular PA | Ridley | 2019 | PA level (range 1-5) | n.s. | n.s. |
|  |  |  | Perceived barrier of lack of time (C)(P) | D'Haese | 2016 | Steps (n/day) | n.s. G (C)(P) / - B (C) / n.s. B (P) | unclear |
|  |  |  | Perceived barrier of lack of transportation to sport activities (C)(P) | D'Haese | 2016 | Steps (n/day) | n.s. G (C)(P) / n.s. B (C) / - B (P) | unclear |
|  |  |  | Perceived barrier of not being allowed to sport (C)(P) | D'Haese | 2016 | Steps (n/day) | n.s. | n.s. |
|  |  |  | Perceived barrier of not being good at sports (C)(P) | D'Haese | 2016 | Steps (n/day) | n.s. | n.s. |
|  |  |  | Perceived barrier of not liking sports (C)(P) | D'Haese | 2016 | Steps (n/day) | n.s. | n.s. |
|  |  |  | Perceived barriers (C) | Pate | 2019a | PA (min/hr) | n.s. | n.s. |
|  |  |  | Don't like PA feel | Ridley | 2019 | PA level (range 1-5) | n.s. | n.s. |
|  | Interpersonal / Social Environmental | Parental influence | Perception of parental support (C)(P) | D'Haese | 2016 | Steps (n/day) | n.s. | n.s. |
|  |  |  | Parental trust in child's ability to be physically active (P) | D'Haese | 2016 | Steps (n/day) | n.s. | n.s. |
|  |  |  | Perception of parental support | Mikalsen | 2020 | MVPA (min/day) | n.s. | n.s. |
|  |  |  | Perceived social norm (C)(P) | D'Haese | 2016 | Steps (n/day) | n.s. | n.s. |
|  |  |  | Perception of parent support (C)(P) | Pate | 2019a | PA (min/hr) | n.s. | n.s. |
|  |  |  | Percention of parent encouragement (C) | Pate | 2019a | PA (min/hr) | + | + |
|  |  |  | Parent's enjoyment of PA (P) | Pate | 2019a | PA (min/hr) | n.s. | n.s. |
|  |  |  | Parent's participation in leisure-time PA (P) | Pate | 2019a | PA (min/hr) | n.s. | n.s. |
|  |  |  | Parent's participation in sports (P) | Pate | 2019a | PA (min/hr) | n.s. | n.s. |
|  |  |  | Importance of child's participation in sports/PA (P) | Pate | 2019a | PA (min/hr) | n.s. | n.s. |
|  |  |  | Rules about sedentary behavior in the home (P) | Pate | 2019a | PA (min/hr) | n.s. | n.s. |
|  |  |  | Parent support | Ridley | 2019 | PA level (range 1-5) | n.s. | n.s. |
|  |  |  | Parent influence: Parent play with | Ridley | 2019 | PA level (range 1-5) | n.s. | n.s. |
|  |  |  | Parent influence: Right clothing | Ridley | 2019 | PA level (range 1-5) | n.s. | n.s. |
|  |  |  | Parent influence: TV Rules imposed by parents | Ridley | 2019 | PA level (range 1-5) | n.s. | n.s. |
|  |  | Peer influence | Peer encouragement CR) | Barr-Anderson | 2017 | Total PA (min/hr) | n.s. | n.s. |
|  |  |  | Friends' co-participation (C) | D'Haese | 2016 | Steps (n/day) | n.s. | n.s. |
|  |  |  | Friend support for PA (C) | Jago | 2012 | After-school MVPA (min/day) | + G / n.s. B | unclear |
|  |  |  |  |  |  | Weekend MVPA (min/day) | + G / n.s. B |  |
|  |  |  | # of friends (C) | Jago | 2012 | After-school MVPA (min/day) | + G / n.s. B | unclear |
|  |  |  |  |  |  | Weekend MVPA (min/day) | + G / n.s. B |  |
|  |  |  | Friend sedentary preferences (C) | Jago | 2012 | After-school MVPA (min/day) | n.s. | n.s. |
|  |  |  |  |  |  | Weekend MVPA (min/day) | n.s. |  |
|  |  |  | General friend support (C) | Jago | 2012 | After-school MVPA (min/day) | n.s. | n.s. |
|  |  |  |  |  |  | Weekend MVPA (min/day) | n.s. |  |
|  |  |  | # of active friends (C) | Pate | 2019a | PA (min/hr) | n.s. | n.s. |
|  |  |  | Peer support (C) | Pate | 2019a | PA (min/hr) | n.s. | n.s. |
|  |  |  | Positive peer influence: Friend play with | Ridley | 2019 | PA level (range 1-5) | n.s | n.s. |
|  |  |  | Positive peer influence: Friends encourage | Ridley | 2019 | PA level (range 1-5) | n.s. | n.s. |
|  |  |  | Barriers associated with peers: Other kids tease | Ridley | 2019 | PA level (range 1-5) | n.s. | n.s. |
|  |  |  | Barriers associated with peers: Bullies | Ridley | 2019 | PA level (range 1-5) | n.s. | n.s. |
|  |  |  | Perceptions of peer-created ego climate | Taylor | 2014 | PA level (range 1-5) | n.s. | n.s. |
|  |  |  | Perceptions of peer-created task climate | Taylor | 2014 | PA level (range 1-5) | n.s. | n.s. |
|  |  | Teacher influence | Perceptions of teacher psychological need support | Taylor | 2014 | PA level (range 1-5) | n.s. | n.s. |
|  | Environmental / Physical Environmental | Home equipment | Acces to active equipment at home (P) | Pate | 2019a | PA (min/hr) | n.s. | n.s. |
|  |  |  | Sedentary equipment in child's bedroom (P) | Pate | 2019a | PA (min/hr) | n.s. | n.s. |
|  |  |  | Sedentary items in home (P) | Pate | 2019a | PA (min/hr) | n.s. | n.s. |
|  |  |  | Availability of PA equipment at home (C) | Pate | 2019a | PA (min/hr) | n.s. | n.s. |
|  |  | Neighborhood environment | Time to closest park (P) | Barr-Anderson | 2017 | Total PA (min/hr) | n.s. | n.s. |
|  |  |  | See children outdoors (P) | Barr-Anderson | 2017 | Total PA (min/hr) | n.s. Black / n.s. White / - Hispanic | unclear |
|  |  |  | Supportiveness of home neigborhood for walking and cycling | Coombes | 2014 | PA level (daily counts per minute) | n.s. | n.s. |
|  |  |  | Convenience of recreational facilities (C)(P) | D'Haese | 2015 | Steps (n/day) | n.s. | n.s. |
|  |  |  | Land use mix diversity (C)(P) | D'Haese | 2015 | Steps (n/day) | n.s. | n.s. |
|  |  |  | Street network connectivity (C)(P) | D'Haese | 2015 | Steps (n/day) | n.s. G (C)(P) / n.s. B (C) / + B (P) | unclear |
|  |  |  | Residential density (C)(P) | D'Haese | 2015 | Steps (n/day) | n.s. | n.s. |
|  |  |  | Land use mix access (C)(P) | D'Haese | 2015 | Steps (n/day) | n.s. | n.s. |
|  |  |  | Availability of walking and cycling infrastructure (C)(P) | D'Haese | 2015 | Steps (n/day) | n.s. | n.s. |
|  |  |  | Maintenance and quality of walking and cycling infrastructure (C)(P) | D'Haese | 2015 | Steps (n/day) | n.s. | n.s. |
|  |  |  | Aesthetics of the neigborhood (C)(P) | D'Haese | 2015 | Steps (n/day) | n.s. | n.s. |
|  |  |  | Traffic safety (C)(P) | D'Haese | 2015 | Steps (n/day) | n.s. | n.s. |
|  |  |  | Crime safety (C)(P) | D'Haese | 2015 | Steps (n/day) | n.s. | n.s. |
|  |  |  | Safe to play outdoors in the neighborhood (y/n) (P) | Forthofer | 2017 | Total PA (min/hr) | n.s. | n.s. |
|  |  |  | Perceived neighborhood environment (C) | Pate | 2019a | PA (min/hr) | n.s. | n.s. |
|  |  |  | Windshield: Incivilities (e.g., litter, graffiti) | Pate | 2019a | PA (min/hr) | n.s. | n.s. |
|  |  |  | Windshield: Social spaces (e.g., presence of yards) | Pate | 2019a | PA (min/hr) | + | + |
|  |  |  | Windshield: Territoriality (e.g., fences or barriers) | Pate | 2019a | PA (min/hr) | n.s. | n.s. |
|  |  |  | Neighborhood Socioeconomic Deprivation | Clennin | 2019 | PA (min/hr) | n.s. | n.s. |
|  |  |  | PARA-index | Pate | 2019a | PA (min/hr) | n.s. | n.s. |
|  |  | School environment | School commute environment supportiveness for walking and cycling | Coombes | 2014 | PA level (daily counts per minute) | n.s. | n.s. |
|  |  |  | Extracurricular PA promotion: Active commuting to school (S) | De Meester | 2014 | Total PA level (mean min/day) | n.s. | n.s. |
|  |  |  |  |  |  | Pedometer/accelerometer weekday steps (mean n/day) | n.s. |  |
|  |  |  |  |  |  | Accelerometer weekday MVPA (mean min/day) | n.s. |  |
|  |  |  | Extracurricular PA promotion: Active school yards or playgrounds (S) | De Meester | 2014 | Total PA level (mean min/day) | n.s. | unclear |
|  |  |  |  |  |  | Pedometer/accelerometer weekday steps (mean n/day) | + |  |
|  |  |  |  |  |  | Accelerometer weekday MVPA (mean min/day) | n.s. |  |
|  |  |  | Extracurricular PA promotion: Health education policy (S) | De Meester | 2014 | Total PA level (mean min/day) | n.s. | n.s. |
|  |  |  |  |  |  | Pedometer/accelerometer weekday steps (mean n/day) | n.s. |  |
|  |  |  |  |  |  | Accelerometer weekday MVPA (mean min/day) | n.s. |  |
|  |  |  | Extracurricular PA promotion: Sports and PA after school (S) | De Meester | 2014 | Total PA level (mean min/day) | n.s. | n.s. |
|  |  |  |  |  |  | Pedometer/accelerometer weekday steps (mean n/day) | n.s. |  |
|  |  |  |  |  |  | Accelerometer weekday MVPA (mean min/day) | n.s. |  |
|  |  |  | Extracurricular PA promotion: Sports and PA during lunch break (S) | De Meester | 2014 | Total PA level (mean min/day) | n.s. | n.s. |
|  |  |  |  |  |  | Pedometer/accelerometer weekday steps (mean n/day) | n.s. |  |
|  |  |  |  |  |  | Accelerometer weekday MVPA (mean min/day) | n.s. |  |
|  |  |  | Changing school | Marks | 2015 | MVPA (min/day) | n.s. | n.s. |
|  |  |  |  |  |  | LPA (min/day) | n.s. |  |
|  |  |  | Intramural activities (S) | Pate | 2019a | PA (min/hr) | - | - |
|  |  |  | Physical education minutes per year (S) | Pate | 2019a | PA (min/hr) | n.s. | n.s. |
|  |  |  | Recess minutes per week (S) | Pate | 2019a | PA (min/hr) | n.s. | n.s. |
| Leisure-time | Individual | Demographic | Gender | De Baere | 2015b | Sport (min/day) | n.s. | n.s. |
|  |  |  |  |  |  | Active leisure (min/day) | n.s. |  |
|  |  |  | Gender | Rutten | 2014 | PA level during leisure time (range 1-5) | n.s. | n.s. |
|  |  | Physical characteristics | BMI: Overweight | Rutten | 2014 | PA level during leisure time (range 1-5) | - | - |
|  |  | Motivation and goals | Child's attitude toward PA (C)(P) | D'Haese | 2016 | Sports during leisure (min/day) | n.s. | n.s. |
|  |  | Beliefs about capabilities | Self-efficacy | D'Haese | 2016 | Sports during leisure (min/day) | + G (C) / n.s. G (P) / n.s. B (C)(P) | unclear |
|  |  | Beliefs about consequences | Perceived benefit of being better than others (C)(P) | D'Haese | 2016 | Sports during leisure (min/day) | n.s. | n.s. |
|  |  |  | Perceived benefit of fun (C)(P) | D'Haese | 2016 | Sports during leisure (min/day) | n.s. | n.s. |
|  |  |  | Perceived benefit of health (C)(P) | D'Haese | 2016 | Sports during leisure (min/day) | n.s. | n.s. |
|  |  |  | Perceived benefit of meeting (new) friends (C)(P) | D'Haese | 2016 | Sports during leisure (min/day) | n.s. | n.s. |
|  |  |  | Perceived benefit of not feeling bored (C)(P) | D'Haese | 2016 | Sports during leisure (min/day) | n.s. | n.s. |
|  |  |  | Perceived benefit of weight loss (C)(P) | D'Haese | 2016 | Sports during leisure (min/day) | n.s. | n.s. |
|  |  |  | Perceived barrier of lack of time (C)(P) | D'Haese | 2016 | Sports during leisure (min/day) | n.s. | n.s. |
|  |  |  | Perceived barrier of lack of transportation to sport activities (C)(P) | D'Haese | 2016 | Sports during leisure (min/day) | n.s. | n.s. |
|  |  |  | Perceived barrier of not being allowed to sport (C)(P) | D'Haese | 2016 | Sports during leisure (min/day) | n.s. | n.s. |
|  |  |  | Perceived barrier of not being good at sports (C)(P) | D'Haese | 2016 | Sports during leisure (min/day) | n.s. | n.s. |
|  |  |  | Perceived barrier of not liking sports (C)(P) | D'Haese | 2016 | Sports during leisure (min/day) | n.s. G (C)(P) / n.s. B (C) / - B (P) | unclear |
|  | Interpersonal / Social Environmental | Parental influence | Perception of parental support (C)(P) | D'Haese | 2016 | Sports during leisure (min/day) | n.s. G (C)(P) / + B (C) / n.s. B (P) | unclear |
|  |  |  | Parental trust in child's ability to be physically active (P) | D'Haese | 2016 | Sports during leisure (min/day) | n.s. | n.s. |
|  |  |  | Perceived social norm (C)(P) | D'Haese | 2016 | Sports during leisure (min/day) | n.s. | n.s. |
|  |  | Peer influence | Friends' co-participation (C) | D'Haese | 2016 | Sports during leisure (min/day) | n.s. | n.s. |
|  | Environmental / Physical Environmental | Neighborhood environment | Convenience of recreational facilities (C)(P) | D'Haese | 2015 | Sports during leisure (min/day) | - G (C) / n.s. G (P) / n.s. B (C)(P) | unclear |
|  |  |  | Land use mix diversity (C)(P) | D'Haese | 2015 | Sports during leisure (min/day) | n.s. | n.s. |
|  |  |  | Street network connectivity (C)(P) | D'Haese | 2015 | Sports during leisure (min/day) | n.s. | n.s. |
|  |  |  | Residential density (C)(P) | D'Haese | 2015 | Sports during leisure (min/day) | n.s. | n.s. |
|  |  |  | Land use mix access (C)(P) | D'Haese | 2015 | Sports during leisure (min/day) | n.s. | n.s. |
|  |  |  | Availability of walking and cycling infrastructure (C)(P) | D'Haese | 2015 | Sports during leisure (min/day) | n.s. | n.s. |
|  |  |  | Maintenance and quality of walking and cycling infrastructure (C)(P) | D'Haese | 2015 | Sports during leisure (min/day) | n.s. | n.s. |
|  |  |  | Aesthetics of the neigborhood (C)(P) | D'Haese | 2015 | Sports during leisure (min/day) | n.s. | n.s. |
|  |  |  | Traffic safety (C)(P) | D'Haese | 2015 | Sports during leisure (min/day) | n.s. | n.s. |
|  |  |  | Crime safety (C)(P) | D'Haese | 2015 | Sports during leisure (min/day) | n.s. | n.s. |
|  |  | School environment | Changing school | Marks | 2015 | After-school being very active (min/day) | n.s. | n.s. |
|  |  |  |  |  |  | Weekend being very active (min/day) | n.s. |  |
| Transport | Individual | Demographic | Gender | De Baere | 2015b | Active travel (min/day) | n.s. | n.s. |
|  |  |  | Gender (C) | De Meester | 2014 | Active transport to school (mean min/day) | n.s. | n.s. |
|  |  |  | Socioeconomic status by parent education | Vanwolleghem | 2016 | Active transport to school (maintaining/switching to passive/active) | n.s. | n.s. |
|  |  |  |  |  |  | Active transport to leisure time destinations (maintaining/switching to passive/active) | n.s. |  |
|  |  | Motivation and goals | Child's attitude toward PA (C)(P) | D'Haese | 2016 | Active transport to school (min/day) | n.s. | n.s. |
|  |  |  |  |  |  | Walking for transport during leisure (min/day) | n.s. |  |
|  |  |  |  |  |  | Cycling for transport during leisure (min/day) | n.s. |  |
|  |  | Beliefs about capabilities | Self-efficacy (C) | D'Haese | 2016 | Active transport to school (min/day) | n.s. | n.s. |
|  |  |  |  |  |  | Walking for transport during leisure (min/day) | n.s. |  |
|  |  |  |  |  |  | Cycling for transport during leisure (min/day) | n.s. |  |
|  |  | Beliefs about consequences | Perceived benefit of being better than others (C)(P) | D'Haese | 2016 | Active transport to school (min/day) | n.s. G (C) / + G (P) / n.s. B (C)(P) | unclear |
|  |  |  |  |  |  | Walking for transport during leisure (min/day) | n.s. |  |
|  |  |  |  |  |  | Cycling for transport during leisure (min/day) | n.s. |  |
|  |  |  | Perceived benefit of fun (C)(P) | D'Haese | 2016 | Active transport to school (min/day) | n.s. | n.s. |
|  |  |  |  |  |  | Walking for transport during leisure (min/day) | n.s. |  |
|  |  |  |  |  |  | Cycling for transport during leisure (min/day) | n.s. |  |
|  |  |  | Perceived benefit of health (C)(P) | D'Haese | 2016 | Active transport to school (min/day) | + G (C) / n.s. G (P) / n.s. B (C)(P) | unclear |
|  |  |  |  |  |  | Walking for transport during leisure (min/day) | n.s. |  |
|  |  |  |  |  |  | Cycling for transport during leisure (min/day) | n.s. |  |
|  |  |  | Perceived benefit of meeting (new) friends (C)(P) | D'Haese | 2016 | Active transport to school (min/day) | n.s. | n.s. |
|  |  |  |  |  |  | Walking for transport during leisure (min/day) | n.s. |  |
|  |  |  |  |  |  | Cycling for transport during leisure (min/day) | n.s. |  |
|  |  |  | Perceived benefit of not feeling bored (C)(P) | D'Haese | 2016 | Active transport to school (min/day) | n.s. | n.s. |
|  |  |  |  |  |  | Walking for transport during leisure (min/day) | n.s. |  |
|  |  |  |  |  |  | Cycling for transport during leisure (min/day) | n.s. |  |
|  |  |  | Perceived benefit of weight loss (C)(P) | D'Haese | 2016 | Active transport to school (min/day) | n.s. | n.s. |
|  |  |  |  |  |  | Walking for transport during leisure (min/day) | n.s. |  |
|  |  |  |  |  |  | Cycling for transport during leisure (min/day) | n.s. |  |
|  |  |  | Attitude / benefits-barriers (P) | Vanwolleghem | 2016 | Active transport to school (maintaining/switching to passive/active) | n.s. | n.s. |
|  |  |  |  |  |  | Active transport to leisure time destinations (maintaining/switching to passive/active) | n.s. |  |
|  |  |  | Perceived barrier of lack of time (C)(P) | D'Haese | 2016 | Active transport to school (min/day) | n.s. | n.s. |
|  |  |  |  |  |  | Walking for transport during leisure (min/day) | n.s. |  |
|  |  |  |  |  |  | Cycling for transport during leisure (min/day) | n.s. |  |
|  |  |  | Perceived barrier of lack of transportation to sport activities (C)(P) | D'Haese | 2016 | Active transport to school (min/day) | n.s. | n.s. |
|  |  |  |  |  |  | Walking for transport during leisure (min/day) | n.s. |  |
|  |  |  |  |  |  | Cycling for transport during leisure (min/day) | n.s. |  |
|  |  |  | Perceived barrier of not being allowed to sport (C)(P) | D'Haese | 2016 | Active transport to school (min/day) | + G (C) / n.s. G (P) / n.s. B (C)(P) | unclear |
|  |  |  |  |  |  | Walking for transport during leisure (min/day) | n.s. |  |
|  |  |  |  |  |  | Cycling for transport during leisure (min/day) | n.s. |  |
|  |  |  | Perceived barrier of not being good at sports (C)(P) | D'Haese | 2016 | Active transport to school (min/day) | n.s. | n.s. |
|  |  |  |  |  |  | Walking for transport during leisure (min/day) | n.s. |  |
|  |  |  |  |  |  | Cycling for transport during leisure (min/day) | n.s. |  |
|  |  |  | Perceived barrier of not liking sports (C)(P) | D'Haese | 2016 | Active transport to school (min/day) | n.s. | unclear |
|  |  |  |  |  |  | Walking for transport during leisure (min/day) | n.s. G (C)(P) / n.s. B (C) / + B (P) |  |
|  |  |  |  |  |  | Cycling for transport during leisure (min/day) | n.s. |  |
|  | Interpersonal / Social Environmental | Parental influence | Perception of parental support (C)(P) | D'Haese | 2016 | Active transport to school (min/day) | n.s. | unclear |
|  |  |  |  |  |  | Walking for transport during leisure (min/day) | n.s. G (C)(P) / n.s. B (C) / + B (P) |  |
|  |  |  |  |  |  | Cycling for transport during leisure (min/day) | n.s. |  |
|  |  |  | Parental trust in child's ability to be physically active (P) | D'Haese | 2016 | Active transport to school (min/day) | n.s. | n.s. |
|  |  |  |  |  |  | Walking for transport during leisure (min/day) | n.s. |  |
|  |  |  |  |  |  | Cycling for transport during leisure (min/day) | n.s. |  |
|  |  |  | Perceived social norm (C)(P) | D'Haese | 2016 | Active transport to school (min/day) | n.s. | n.s. |
|  |  |  |  |  |  | Walking for transport during leisure (min/day) | n.s. |  |
|  |  |  |  |  |  | Cycling for transport during leisure (min/day) | n.s. |  |
|  |  | Peer influence | Friends' co-participation (C) | D'Haese | 2016 | Active transport to school (min/day) | n.s. | n.s. |
|  |  |  |  |  |  | Walking for transport during leisure (min/day) | n.s. |  |
|  |  |  |  |  |  | Cycling for transport during leisure (min/day) | n.s. |  |
|  | Environmental / Physical Environmental | Neighborhood environment | Supportiveness of home neigborhood for walking and cycling | Coombes | 2014 | Travel mode to school (passive/active) | n.s. | n.s. |
|  |  |  | Convenience of recreational facilities (C)(P) | D'Haese | 2015 | Active transport to school (min/day) | n.s. | n.s. |
|  |  |  |  |  |  | Walking for transport during leisure (min/day) | n.s. |  |
|  |  |  |  |  |  | Cycling for transport during leisure (min/day) | n.s. |  |
|  |  |  | Land use mix diversity (C)(P) | D'Haese | 2015 | Active transport to school (min/day) | n.s. | n.s. |
|  |  |  |  |  |  | Walking for transport during leisure (min/day) | n.s. |  |
|  |  |  |  |  |  | Cycling for transport during leisure (min/day) | n.s. |  |
|  |  |  | Street network connectivity (C)(P) | D'Haese | 2015 | Active transport to school (min/day) | n.s. | n.s. |
|  |  |  |  |  |  | Walking for transport during leisure (min/day) | n.s. |  |
|  |  |  |  |  |  | Cycling for transport during leisure (min/day) | n.s. |  |
|  |  |  | Residential density (C)(P) | D'Haese | 2015 | Active transport to school (min/day) | n.s. | n.s. |
|  |  |  |  |  |  | Walking for transport during leisure (min/day) | n.s. |  |
|  |  |  |  |  |  | Cycling for transport during leisure (min/day) | n.s. |  |
|  |  |  | Land use mix access (C)(P) | D'Haese | 2015 | Active transport to school (min/day) | n.s. | n.s. |
|  |  |  |  |  |  | Walking for transport during leisure (min/day) | n.s. |  |
|  |  |  |  |  |  | Cycling for transport during leisure (min/day) | n.s. |  |
|  |  |  | Availability of walking and cycling infrastructure (C)(P) | D'Haese | 2015 | Active transport to school (min/day) | n.s. | unclear |
|  |  |  |  |  |  | Walking for transport during leisure (min/day) | n.s. |  |
|  |  |  |  |  |  | Cycling for transport during leisure (min/day) | n.s. G (C) / + G (P) / n.s. B (C) / - B (P) |  |
|  |  |  | Maintenance and quality of walking and cycling infrastructure (C)(P) | D'Haese | 2015 | Active transport to school (min/day) | n.s. | n.s. |
|  |  |  |  |  |  | Walking for transport during leisure (min/day) | n.s. |  |
|  |  |  |  |  |  | Cycling for transport during leisure (min/day) | n.s. |  |
|  |  |  | Aesthetics of the neigborhood (C)(P) | D'Haese | 2015 | Active transport to school (min/day) | n.s. | n.s. |
|  |  |  |  |  |  | Walking for transport during leisure (min/day) | n.s. |  |
|  |  |  |  |  |  | Cycling for transport during leisure (min/day) | n.s. |  |
|  |  |  | Traffic safety (C)(P) | D'Haese | 2015 | Active transport to school (min/day) | n.s. | n.s. |
|  |  |  |  |  |  | Walking for transport during leisure (min/day) | n.s. |  |
|  |  |  |  |  |  | Cycling for transport during leisure (min/day) | n.s. |  |
|  |  |  | Crime safety (C)(P) | D'Haese | 2015 | Active transport to school (min/day) | n.s. | unclear |
|  |  |  |  |  |  | Walking for transport during leisure (min/day) | n.s. |  |
|  |  |  |  |  |  | Cycling for transport during leisure (min/day) | n.s. G (C)(P) / - B (C) / n.s. B (P) |  |
|  |  | School environment | School commute environment supportiveness for walking and cycling | Coombes | 2014 | Travel mode to school (passive/active) | n.s | n.s. |
|  |  |  | Extracurricular PA promotion: Active commuting to school (S) | De Meester | 2014 | Active transport to school (mean min/day) | n.s. | n.s. |
|  |  |  | Extracurricular PA promotion: Active school yards or playgrounds (S) | De Meester | 2014 | Active transport to school (mean min/day) | n.s. | n.s. |
|  |  |  | Extracurricular PA promotion: Health education policy (S) | De Meester | 2014 | Active transport to school (mean min/day) | n.s. | n.s. |
|  |  |  | Extracurricular PA promotion: Sports and PA after school (S) | De Meester | 2014 | Active transport to school (mean min/day) | n.s. | n.s. |
|  |  |  | Extracurricular PA promotion: Sports and PA during lunch break (S) | De Meester | 2014 | Active transport to school (mean min/day) | n.s. | n.s. |
|  |  |  | Changing school | Marks | 2015 | Walking to school (times/wk) | n.s. | unclear |
|  |  |  |  |  |  | Cycle/scoot to school (times/wk) | - |  |
|  |  |  | Difference home-school distance between primary and secondary school | Vanwolleghem | 2016 | Active transport to school (maintaining/switching to passive/active) | - | unclear |
|  |  |  |  |  |  | Active transport to leisure time destinations (maintaining/switching to passive/active) | n.s. |  |
| School | Individual | Demographic | Gender | De Baere | 2015b | School (min/day) | n.s. | n.s. |
|  |  |  | Gender (C) | De Meester | 2014 | Extracurricular PA (min/day) | n.s. | n.s. |
|  |  |  | Gender | Rutten | 2014 | PA level at school (range 1-5) | n.s. | n.s. |
|  |  |  | Socioeconomic status | Shin | 2019 | Perceived physical education activity (range 1-5) | n.s. | n.s. |
|  |  | Physical characteristics | BMI: Overweight | Rutten | 2014 | PA level at school (range 1-5) | n.s. | n.s. |
|  |  | Self-concept | Perceived Aggressive Disposition | Shin | 2019 | Perceived physical education activity (range 1-5) | n.s. | n.s. |
|  | Interpersonal / Social Environmental | Peer influence | Perceived Peer Acceptance | Shin | 2019 | Perceived physical education activity (range 1-5) | n.s. | n.s. |
|  | Environmental / Physical Environmental | School environment | Extracurricular PA promotion: Active commuting to school (S) | De Meester | 2014 | Extracurricular PA (min/day) | n.s. | n.s. |
|  |  |  | Extracurricular PA promotion: Active school yards or playgrounds (S) | De Meester | 2014 | Extracurricular PA (min/day) | n.s. | n.s. |
|  |  |  | Extracurricular PA promotion: Health education policy (S) | De Meester | 2014 | Extracurricular PA (min/day) | + | + |
|  |  |  | Extracurricular PA promotion: Sports and PA after school (S) | De Meester | 2014 | Extracurricular PA (min/day) | n.s. | n.s. |
|  |  |  | Extracurricular PA promotion: Sports and PA during lunch break (S) | De Meester | 2014 | Extracurricular PA (min/day) | n.s. | n.s. |
|  |  |  | Changing school | Marks | 2015 | Being very active in physical education class (frequency) | n.s. | unclear |
|  |  |  |  |  |  | Do most of the time at recess | - |  |
|  |  |  |  |  |  | Do most of the time at lunch | - |  |
| Home | Individual | Demographic | Gender | De Baere | 2015b | CADL (min/day) | n.s. | n.s. |

C = child-reported variable; P = parent-reported variable; S = school-reported variable; PA = physical activity; LPA = light physical activity; MVPA = moderate-to-vigorous physical activity; CADL = common activities of daily life; G = girls; B = boys. Association is reported as not significant (n.s.), positive/protective against declines (+), negative/contributing to declines (-) or unclear.

Table S4. Summary of reasons to report associations as unclear

|  |  | **Domain** | | | | **Reason for unclear** | | | | |
| --- | --- | --- | --- | --- | --- | --- | --- | --- | --- | --- |
| **Sample** | **Author (year)** | **General** | **Leisure-time** | **Transport** | **School** | **1** | **2** | **3** | **4** | **5** |
| TRACK | Barr-Anderson (2017) | 2 |  |  |  |  | 2 |  |  |  |
| De Meester (2014) | De Meester (2014) | 1 |  |  |  | 1 |  |  |  |  |
|  | D'Haese (2015) | 1 | 2 | 2 |  |  |  |  | 3 | 2 |
|  | D'Haese (2016) | 3 | 2 | 5 |  |  |  |  | 5 | 5 |
|  | Vanwolleghem (2016) |  |  | 1 |  | 1 |  |  |  |  |
| PEACH | Jago (2012) | 2 |  |  |  |  |  | 2 |  |  |
|  | Marks (2015) |  |  | 1 | 1 | 2 |  |  |  |  |

TRACK = Transitions and Activity Changes in Kids; PEACH = Personal and Environmental Associations with Children’s Health; Reasons for unclear: 1 = Differences in results between PA outcomes; 2 = Differences in results between subgroups (i.e., race/ethnicity); 3 = Differences in results between subgroups (i.e., sex/gender); 4 = Differences in results between subgroups (i.e., sex/gender) and child-/parent-reported variable; 5 = Combination of differences in results between PA outcomes, subgroups (i.e., gender) and child-/parent-reported variable.
